# Regulation of Hfq mRNA and Protein Levels in *Escherichia coli* and *Pseudomonas aeruginosa* by the *Burkholderia cenocepacia* MtvR sRNA

**DOI:** 10.1371/journal.pone.0098813

**Published:** 2014-06-05

**Authors:** Christian G. Ramos, André M. Grilo, Sílvia A. Sousa, Joana R. Feliciano, Paulo J. P. da Costa, Jorge H. Leitão

**Affiliations:** Department of Bioengineering and Institute for Biotechnology and Bioengineering, Instituto Superior Técnico, Universidade de Lisboa, Lisboa, Portugal; Ghent University, Belgium

## Abstract

Small non-coding RNAs (sRNAs) are important players of gene expression regulation in bacterial pathogens. MtvR is a 136-nucleotide long sRNA previously identified in the human pathogen *Burkholderia cenocepacia* J2315 and with homologues restricted to bacteria of the *Burkholderia cepacia* complex. In this work we have investigated the effects of expressing MtvR in *Escherichia coli* and *Pseudomonas aeruginosa*. Results are presented showing that MtvR negatively regulates the *hfq* mRNA levels in both bacterial species. In the case of *E. coli*, this negative regulation is shown to involve binding of MtvR to the 5′-UTR region of the *hfq*_Ec_ mRNA. Results presented also show that expression of MtvR in *E. coli* and *P. aeruginosa* originates multiple phenotypes, including reduced resistance to selected stresses, biofilm formation ability, and increased susceptibility to various antibiotics.

## Introduction

Small non-coding RNAs (sRNAs) are increasingly recognized as important players in gene expression regulation in bacteria, and more recently, in the regulation of virulence determinants in bacterial pathogens [1]. Many of the sRNAs characterized so far exert their action by base-pairing with their target mRNAs in the region of the ribosome-binding site, thus affecting their stability and/or translation [2]. This mode of regulation has been shown to be advantageous for bacteria when a fast response to external stimuli is required, providing a fine tuning of gene expression [3]. This also enables the bacterial cell with a precise gene expression regulation and the means for a rapid adaptation in response to specific environmental changes [4]. These advantages are consistent with the involvement of many described bacterial sRNAs in response to stress [5]. In addition, sRNAs and the associated complex regulatory circuits are also used by bacterial pathogens to adapt to the host environment, and to coordinately express specific virulence determinants during different stages of infection [6].

*Trans*-encoded sRNAs are arguably the most extensively characterized class of bacterial sRNAs [6]. These sRNAs can target one or more mRNAs, sharing with them a limited complementarity. In order to interact with their targets, most of the *trans*-encoded sRNAs require the aid of the RNA chaperone Hfq, which plays a role as a facilitator of sRNA-mRNA interactions [7].

Most of the sRNAs recently described are only conserved among closely related bacterial species [8]. This is the case of MtvR, a 136 nucleotide-long sRNA identified in *Burkholderia cenocepacia* J2315, with homologues restricted to bacteria of the *Burkholderia* genus [9]. *B. cenocepacia* is a member of the so called *Burkholderia cepacia* complex (Bcc), a group of closely related species that emerged in the 1980s as important opportunistic pathogens among patients afflicted with the genetic disease cystic fibrosis [10], and more recently, among hospitalized non-cystic fibrosis patients, especially cancer patients (reviewed in [11]).

Previous work from our research group has shown that the sRNA MtvR targets multiple genes in the clinical isolate *B. cenocepacia* J2315, affecting cell size, growth and survival under nutrient deprivation, biofilm formation, antibiotics resistance, and virulence to the nematode *Caenorhabditis elegans* [9]. Due to the pleiotropic phenotypes observed, MtvR was proposed as a global regulatory sRNA in *B. cenocepacia* [9]. *hfq* was previously shown as one of the MtvR multiple targets in *B. cenocepacia*, with the sRNA negatively regulating *hfq* translation by specifically binding to its 5′ leader region [9]. In the present study, we investigated the possible roles played by MtvR in the non-Bcc organisms *Escherichia coli* and *Pseudomonas aeruginosa*, two bacterial species with no MtvR homologues, but harboring Hfq-encoding genes. Despite being an exogenous sRNA in both bacterial species, results here presented indicate that MtvR modulates the levels of the Hfq mRNA and protein in both species, affecting their resistance to selected stresses, including to antibiotics.

## Materials and Methods

### Bacterial Strains, Culture Conditions, Plasmids and Primers

Bacterial strains and plasmids are described in Table 1, and oligonucleotides are listed in Table 2. *P. aeruginosa* strains were maintained on PIA (Pseudomonas Isolation Agar, Becton Dickinson) plates, supplemented with 650 µg.ml^−1^ trimethoprim, when appropriate. *E. coli* strains were maintained on Lennox Broth (LB) plates supplemented with 100 µg.ml^−1^ trimethoprim or 50 µg.ml^−1^ kanamycin or chloramphenicol, when appropriate. Unless otherwise stated, liquid cultures were carried out using LB liquid medium, supplemented with antibiotics when appropriate, with orbital agitation (250 rev min^−1^) at 37°C. Expression of MtvR in *E. coli* or *P. aeruginosa* was achieved by induction of plasmid pCGR12 (Table 1) with 0.1% (v/v) L-arabinose. Plasmid pCGR34 was derived from pCR II by cloning into the XbaI-HindIII sites the *E. coli* MC4100 *hfq* (*hfq*_Ec_) coding sequence, including the complete 5′ leader region and a 6×histidine tag encoded at the C-terminus. Plasmid pCGR35, containing the genetic fusion composed of the *hfq*_Ec_ leader -β-galactosidase, was engineered as follows: the LacZα fragment was amplified by PCR from pBBR1MCS and blunt-end ligated to the EcoRV site of pMLBAD. A 380 bp fragment containing the 5′-UTR region of the *hfq*_Ec_ gene was obtained by XbaI-BamHI restriction of a PCR fragment obtained using as template *E. coli* MC4100 chromosomal DNA. After restriction, the fragment was filled-in with the Klenow large fragment of DNA polymerase I (Invitrogen), and then blunt-ligated to the filled-in XbaI site of the cloned LacZα fragment. Plasmid pCGR36 derives from pCR II, and contains the *hfq*_Ec_ 5′ leader region together with the coding sequence in the XbaI-HindIII sites, allowing T7-driven transcription. Plasmid pCGR37 derives from pMLBAD, and contains the 255 nt fragment corresponding to the *P. aeruginosa* PA14 *hfq* (*hfq*_Pa_, obtained by PCR using as template *P. aeruginosa* PA14 chromosomal DNA), cloned in the PstI-SalI sites (3′ to 5′ direction of the coding sequence). Plasmid pCGR38 derives from pMLBAD and contains the 140 bp cDNA fragment corresponding to the MtvR sRNA cloned in the EcoRI/XbaI sites and the 255 bp cDNA fragment corresponding to the *P. aeruginosa* PA14 *hfq* cloned in the SalI/PstI sites (tandem cloning, pBAD promoter control). All plasmid constructions were verified by DNA sequencing.

**Table 1:** Bacterial strains and plasmids used in this work.

Strain or plasmid	Description	Reference or source
*P. aeruginosa* WT	*P. aeruginosa* PA14, clinical isolate	[37]
*P. aeruginosa* WT-*hfq^sil^*	*P. aeruginosa* PA14 with *hfq*_Pa_ silenced after transformationwith pCGR37	This study
*P. aeruginosa* WT/p*hfq*	*P. aeruginosa* PA14 expressing heterologous *hfq*_Bc_ aftertransformation with pSAS3	This study
*P. aeruginosa* WT/p*hfq*+MtvR	*P. aeruginosa* PA14 expressing heterologous *hfq*_Bc_ andMtvR after transformation with pSAS3 and pCGR12	This study
*P. aeruginosa* WT/pMLBAD	*P. aeruginosa* PA14 after transformation with pMLBAD	This study
*P. aeruginosa* WT/MtvR	*P. aeruginosa* PA14 expressing MtvR after transformationwith pCGR12	This study
*P. aeruginosa* WT-*hfq^sil^*/MtvR	*P. aeruginosa* PA14 with *hfq*_Pa_ silenced and expressing MtvR,after transformation with pCGR38	This study
*E. coli* WT	*E. coli* MC4100, laboratory strain	[38]
*E. coli* Δ*hfq*	*E. coli* GS081, Cm^R^	[39]
*E. coli* WT/p*hfq*	*E. coli* MC4100 after transformation with pCGR34, expressingtagged *hfq*_Ec_	This study
*E. coli* WT/p*hfq*+MtvR	*E. coli* MC4100 after transformation with pCGR34 and pCGR12,expressing tagged *hfq*_Ec_ and MtvR	This study
*E. coli* WT/pMLBAD	*E. coli* MC4100 after transformation with pMLBAD	This study
*E. coli* WT/MtvR	*E. coli* MC4100 after transformation with pCGR12,expressing MtvR	This study
*E. coli* Δ*hfq*/pMLBAD	*E. coli* GS081 transformed with pMLBAD	This study
*E. coli* Δ*hfq*/p*hfq*	*E. coli* GS081 transformed with pSAS3, expressingheterologous *hfq*_Bc_	This study
*E. coli* Δ*hfq*/MtvR	*E. coli* GS081 transformed with pCGR12, expressing MtvR	This study
*E. coli* Δ*hfq*/p*hfq*+MtvR	*E. coli* GS081 transformed with pCGR34 and pCGR12,expressing tagged *hfq*_Ec_ and MtvR	This study
	Plasmids	
pCR II	Amp^R^; Km^R^; used for generating *in vitro* transcription templates	Invitrogen
pMLBAD	Tmp^R^; used for inducible gene expression	[40]
pCGR4	pET23a+ with the *hfq*_Bc_ encoding sequence cloned	[14]
pSAS3	pMLBAD with the *hfq*_Bc_ encoding sequence cloned	[14]
pCGR12	pMLBAD with the 140 bp cDNA fragment corresponding tothe MtvR sRNA cloned in the EcoRI/XbaI sites(pBAD promoter control)	[9]
pCGR34	pCR II with the 609 bp cDNA fragment corresponding tothe *E. coli* MC4100 *hfq* full mRNA (5′-UTR and CDS) with6 histidines at the C-terminus, cloned in the XbaI/HindIIIsites (T7 promoter control)	This study
pCGR35	pMLBAD with the *hfq*_Ec_ 5′-UTR-LacZ DNA fragment (pBABpromoter disrupted, only replicative)	This study
pCGR36	pCR II with the DNA fragment corresponding to *E. coli* MC4100*hfq* cloned in the XbaI/HindIII sites	This study
pCGR37	pMLBAD with the 255 bp cDNA fragment corresponding to the*P. aeruginosa* PA14 *hfq* cloned in the PstI/SalI sites	This study
pCGR38	pMLBAD with the 140 bp cDNA fragment corresponding tothe MtvR sRNA cloned in the EcoRI/XbaI sites and the255 bp cDNA fragment corresponding to the *P. aeruginosa*PA14 *hfq* cloned in the SalI/PstI sites(tandem cloning, pBAD promoter control)	This study

**Table 2:** Oligonucleotides and primers used in this work.

Name	Purpose	Sequence 5′ –3′	Source or reference
UF	Cloning mtvR	TTTCTAGATATTGACGGCGGCGGGT	[9]
LF	Cloning mtvR	TTAAGCTTAAATTATAGCGCCCCAATTA	[9]
NP	Northern analysis of MtvR	CTATCACCCGCCTGTGTCGCCA	[9]
HFQ	Northern blot probe for *hfq*	AAAGGGCAATTGTTACAAG	[12]
CGRO117	Amplification of *P. aeruginosa hfq*	TTCTGCAGACCGGACGGCTCGGTACCAC	This study
CGRO118	Amplification of *P. aeruginosa hfq*	TCTGTCGACTTCCGGAGCGAGACCGGAGT	This study
CGRO119	Amplification of *E. coli hfq*	CCTCTAGACCAGAACAGGCGCGTGACGA	This study
CGRO120	Amplification of *E. coli hfq*	TTAAGCTTGAAACCGGGCGAGACGGGAC	This study
CGRO123	Fwd primer *hfq* his-tag	TTTCTAGAGCACGTCCCGCAAGGGCTAG	This study
CGRO124	Rev primer *hfq* his-tag	TTGGATCCATTGTGGTGGTGGTGGTGGGACGAGGCTTCCGC	This study
M13FWD	LacZ amplification	GTAAAACGACGGCCAGT	Invitrogen
M13REV	LacZ amplification	AGCGGATAACAATTTCACACAGGA	Invitrogen
CGRO125	Fwd primer *uhpA*	CTGGGGCTGGAACCTGATTT	This study
CGRO126	Rev primer *uhpA*	CGCAGCAATGAGTTCATCCG	This study
CGRO127	Fwd primer *uhpT*	TTCCTGCCGTTCATGCTGAT	This study
CGRO128	Rev primer *uhpT*	GAGGCCATAAGATTCCGGGG	This study
5S	Northern blot probe for 5S rRNA	TTCGGGATGGGAAGGGGTGGGA	[12]

### DNA Manipulation Techniques

Total DNA was obtained from the indicated cell cultures using the High pure PCR template preparation kit (Roche). PCR amplification was performed using TaqPlatinum (Invitrogen) DNA polimerase and adequate primers and DNA templates (Table 2). PCR products were purified using the NucleoExtract II kit (Nagel-Machery) as previously described [9]. After nuclease restriction, fragments were directionally cloned into the indicated plasmids. All plasmid constructions were confirmed by DNA sequencing.

### RNA Manipulation Techniques

Total RNA isolation, quantification of RNA concentration and visual quality control, and RNA labeling with biotin were performed as previously described [12].

### Northern Blot Analysis

The expression levels of MtvR, *hfq*_Ec_ or *hfq*_Pa_ were assessed by Northern blot analysis using 2 µg of total RNA and adequate oligonucleotide probes (Table 2), previously labeled with biotin, based on previously described methods [13]. Briefly, RNA samples were separated in 8% TBE-urea pre-cast gels (BioRad), using constant current of 200 mA. RNA was then transferred to a BrightStar plus membrane (Ambion) using the wet transfer system (BioRad), at 100 V for 1h. Samples were probed with adequate biotinylated oligonucleotides at 40°C, for 16 h. Hybridization signals were detected using the BrightStar Biodetect kit (Ambion) and Kodak MX X-ray films. The 5S RNA was used as loading control in all Northern blot experiments. Relative expression was estimated with the ImageJ software suite, using the band intensities of the 5S RNA for normalization.

### RNA Decay Experiments

The RNA decay rate was assessed by Northern blot analysis using 2 µg of total RNA, purified from cells of *E. coli* (Ec) or *P.* *aeruginosa* (Pa), harvested from 24 h-cultures, immediately before (t_0_) or after the addition of rifampicin to induce transcriptional arrest. This antibiotic was used at final concentrations of 250 µg ml^−1^. Aliquots taken after 5, 10, 15 and 20 min (for *hfq*), or 2, 5, 10 and 15 min (for MtvR) of transcription arrest, were processed and analyzed by Northern blot, as described above. RNA decay rates were calculated based on the exponential fit expression: t_1/2_ (min): ae^−bt^, and using the slope of semi-log plots.

### RNA *in vitro* Transcription and Labeling

The DNA templates for *in vitro* transcription of the *hfq*_Ec_ mRNA full transcript, the 5′-UTR of *hfq*_Ec_, the *hfq*_Ec_ coding region (CDS), and the MtvR sRNA were obtained by endonuclease restriction of the appropriate plasmids (Table 1). All RNA transcripts were generated from the T7 promoter, using the MEGAshortscript kit (Ambion). The transcripts MtvR (136 nt), 5′-UTR of *hfq*_Ec_ (155 nt), and the CDS of *hfq*_Ec_ (307 nt) were purified from 8%–7 M urea polyacrylamide gels, while the *hfq*_Ec_ full transcript (462 nt) was purified from a 1% TBE/agarose gel. RNA transcripts were processed and labeled based on previously described methods [13]. Signal intensity was tested using the dot-blot procedure, and detected using the Bright Star Biodetect Kit (Ambion).

### Electrophoretic Mobility Shift Assays

EMSA experiments to assess the binding affinity of MtvR to the *hfq*_Ec_ 5′-UTR, the *hfq*_Ec_ coding region (CDS), and the *hfq*_Ec_ full mRNA, were performed as previously described [12]. Briefly, 25 nM of the MtvR, together with 0, 0.5, 1, 5, 10, 50 or 100 nM of the *hfq*_Ec_ full transcript, or with 0, 5, 10, 25, 50, 75, 100, 150, 200, 250 or 500 nM of the *hfq*_Ec_ CDS, or with 0, 0.5, 1, 5, 10, 50 or 100 nM the 5′-UTR of *hfq*_Ec_, were incubated in 25 µl of RNA binding buffer (20 mM Tris.Cl, pH 8.0, 1 mM DTT, 1 mM MgCl_2_, 10 mM KCl and 10 mM KH_2_PO_4_) [14] for 30 min at 25°C. The ability of 25 nM of the 5′-end labeled (with 11-UTP-biotin) *hfq*_Ec_ 5′-UTR to bind to 50, 100 or 250 nM of *B. cenocepacia* J2315 Hfq_6_ (Hfq_Bc_), of 100 nM of *hfq*_Ec_ full RNA to bind to Hfq_Bc_ (concentrations ranging from 10 to 1000 nM of Hfq_Bc_), or to Hfq_Bc_ (concentrations ranging from 0.5 to 250 nM) in the presence of MtvR (concentrations ranging from 0.1 to 100 nM), were also evaluated by EMSA experiments using native 8% polyacrylamide gel containing 10% glycerol.

Non-labeled yeast tRNA (Ambion) was added in excess to each sample to minimize non-specific binding. Incubation, resolution of RNA-RNA and RNA-protein complexes, and detection of band-shifts, were performed as previously described [14]. The curves generated from the data plots were fitted by non-linear least squares regression assuming a bimolecular model in which the *K*_d_ values represent the protein concentration at half the maximal RNA binding.

### Reverse Transcription-PCR Experiments

Total RNA was extracted from cells of exponentially growing cultures of *E. coli* WT strain, *E. coli* WT expressing MtvR, or *E. coli* Δ*hfq* mutant, using the already described methods. Reverse transcription reactions were performed using the First Strand cDNA synthesis kit (Fermentas), with an incubation of 75 min at 45°C, in a total volume of 50 µL, and 500 ng of total RNA. The cDNA samples were used in PCR experiments with TaqMed (Citomed) DNA polymerase, and the oligonucleotide pairs CGRO125 and CGRO126 (*uhpA*), or CGRO127 and CGRO128 (*uhpT*) (Table 2). Cycling conditions were as follows: 45 cycles of denaturation at 95°C for 1 min, annealing at 55°C for 1 min, extension at 72°C for 0.5 min, and a final extension at 72°C for 10 min. cDNA pools were resolved in 2%-TBE agarose gels. Control reactions using DNA were included. 500 ng of total RNA from each strain were also loaded on the same gel, as loading controls.

### Western Blot Analysis

The effect of MtvR expression on the levels of the Hfq_Ec_ protein was assessed by Western blotting. For this purpose, plasmid pCGR34 was introduced into the *E. coli* Δ*hfq* mutant strain. Plasmid pCGR34 is able to drive the transcription of *hfq*_Ec_ as a derivative containing a 6×His-tag at the C-terminus (Hfq_Ec_-His), from its own promoter. This strain was further transformed with plasmid pCGR12, which allows MtvR expression. The cultures were grown for 16 h in LB liquid medium, supplemented with 150 µg ml^−1^ trimethoprim, 50 µg ml^−1^ kanamycin and 0.1% L-arabinose. Total proteins from culture aliquots of 1 ml were purified with Illustra TriplePrep kit (GE Healthcare), based on previously described methods [13]. Aliquots containing 10 µg of total protein were used for Western blot detection of the Hfq_Ec_-His protein, using the polyclonal pentaHis-IgG-HRP antibody (Invitrogen). The α-GroEL, used as loading control in Western-blot experiments, was detected with a goat anti-GroEL antibody (SicGen, Portugal). Signals were detected using a ECL system (GE Healthcare). Fold-change values were estimated using as unitary value the number of pixels counted for the reference, normalized with the internal standard.

### β-galactosidase Assays

β-galactosidase assays were performed based on previously described methods [15] using a SpectraMax 250 microtiter plate reader (Molecular Devices). Briefly, *E. coli* strains WT, WT transformed with the plasmid that allows MtvR expression, or transformed with the control vector pMLBAD, were grown at 37°C in LB medium for 24 h with antibiotics (when appropriate) and diluted 1000-fold into 50 mL of fresh medium at 37°C. Cultures were grown with agitation to an OD_640_ of 0.1 before inducing MtvR expression by the addition of L-arabinose (0.1% final concentration). Specific β-galactosidase activities (OD_640_ ∼1.0) were calculated using the formula V_max_/OD_600_. The reported results represent data from at least four independent experiments.

### Antibiotic Susceptibility and Biofilm Formation Experiments

The susceptibility of *E. coli* WT, *P. aeruginosa* WT, and of the respective derivatives to the antibiotics chloramphenicol, ciprofloxacin, tetracycline, tobramycin, gentamycin and ampicillin, was assessed by the broth micro-dilution method, in Mueller-Hinton medium (Gibco) supplemented with 0.1% arabinose, based on previously described methods [16], and following the CLSI guidelines [17]. The ability of *E. coli* WT, *P. aeruginosa* WT and the respective derivatives to form biofilms was assessed after 24, 48 and 72-h of growth on LB liquid medium at 28°C, based on previously published methods [18]. Briefly, appropriate volumes of overnight liquid cultures of bacterial strains were used to inoculate LB liquid medium and grown at 28°C with orbital agitation, until the mid-exponential phase was reached. The cultures were subsequently diluted to a standardized culture OD_640_ of 0.5, and 20 µl of these cell suspensions were used to inoculate the wells of a 96-well polystyrene microtiter plate (Greiner Bio-One) containing 180 µl of LB medium. Plates were incubated at 28°C without agitation for 24, 48 and 72 h. The biofilm formed was quantified by measuring the absorbance at 590 nm using a VERSAmax microplate reader (Molecular Devices). All measurements were performed in triplicate, using biological duplicates.

### Growth and Nutrient Deprivation Kinetics

Cultures of *E. coli* WT, *P. aeruginosa* WT and the respective derivatives were carried out at 37°C in liquid LB supplemented with 0.1% L-arabinose. Growth was followed spectrophotometrically at 640 nm. The ability of *E. coli* WT, *P. aeruginosa* WT and respective derivatives to survive to nutrient limiting conditions was assayed in M9 minimal medium supplemented with 0.1% L-arabinose instead of glucose, based on previously described methods [12]. Determination of total CFU’s was performed by plating aliquots of each culture in solid LB supplemented with 0.1% L-arabinose. All measurements were performed in quadruplicate, using biological triplicates.

### Stress Susceptibility Experiments

The susceptibility of *E. coli* WT or *P. aeruginosa* WT and derivative strains to the stresses imposed by growth in LB solid medium containing 0.1% (w/v) L-arabinose, and supplemented with 0.05% SDS, 2.5% (w/v) NaCl, 2.5% (v/v) ethanol, or 25 µM methyl viologen, were performed as previously described [12]. Results are the means of at least 5 independent experiments.

### Statistics Analysis

An unpaired two-tailed chi-square test was used to calculate the *P* values for β-galactosidase assays. Analysis of data from Northern and Western blotting was performed using a paired one-tailed *t* test to calculate the *P* values (P<0.01 [*]; p<0.005 [**]). Error bars represent the means of the standard deviation. Images shown are representative of the experiments performed. All experiments were repeated independently at least 4 times, using biological triplicates, with a minimum *n* value of 12.

### Bioinformatics

BLAST searches were performed using the Integrated Microbial Genomes (IMG) webserver [19], using an E-value ≤1e^−50^ as cut-off. MtvR putative targets were predicted using the sRNATarget [20], the TargetRNA [21], and the RNAPredator [22] programs, with a cut-off of 1, a minimum seed of 7 nucleotides, and an hybridization target region window size of −100 to +30 around the translation start site. The MtvR sRNA sequence was aligned with the RNA sequence of the putative mRNA targets, using the sequences retrieved from NCBI for the *Escherichia coli* str. K12 substr. DH10B and the *Pseudomonas aeruginosa* UCBPP-PA14 genome sequences, using the RNAHybrid web tool [23], using a minimum seed of 7 nucleotides [24] in the region around the start codon.

## Results

### MtvR Regulates the mRNA Levels of *E. coli* and *P.* *aeruginosa hfq* Genes by Promoting their Accelerated Decay

**Figure 1:**
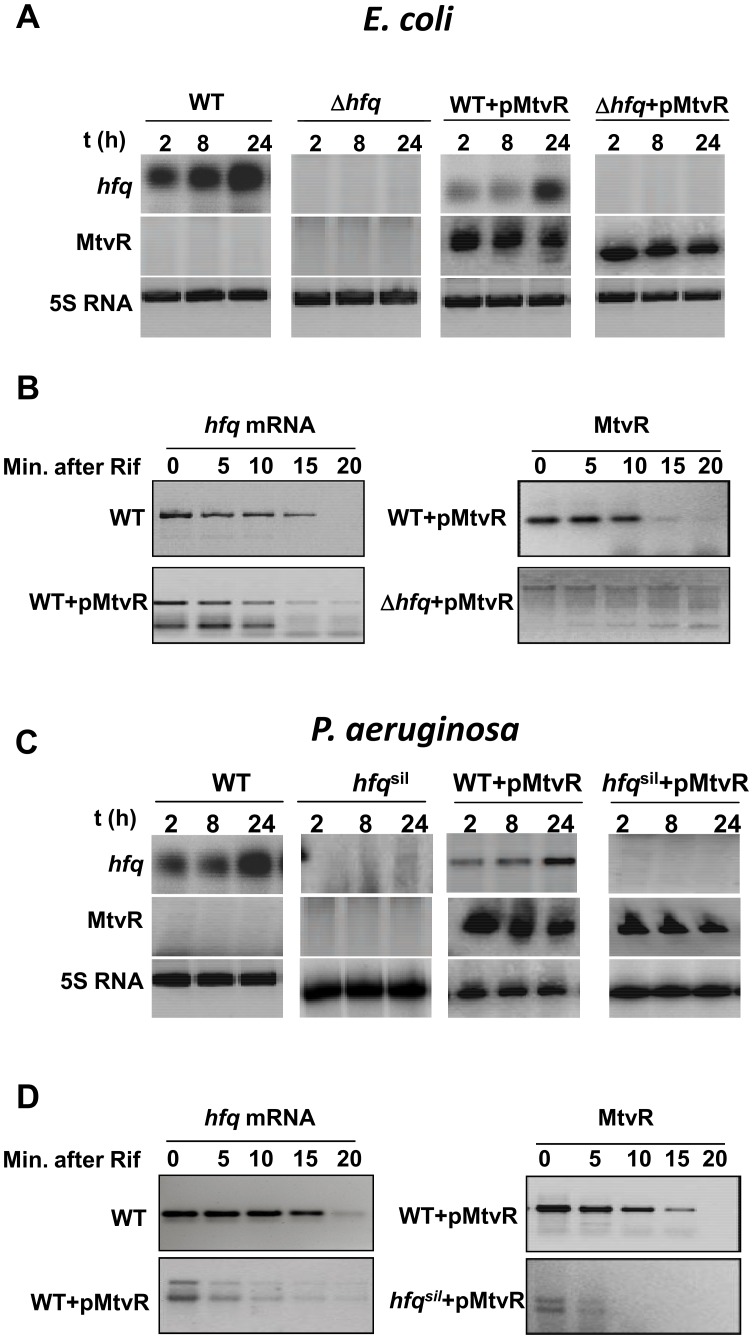
MtvR regulates the mRNA levels of the *E. coli* and *P. aeruginosa hfq* genes. (A) Levels the *hfq_Ec_* mRNA and the MtvR transcript in cells of the *E. coli* WT or the *E. coli* Δ*hfq* mutant, expressing (+pMtvR) or not the sRNA. (B) Stability of the *hfq*_Ec_ mRNA (left panel) in cells expressing (+pMtvR) or not MtvR, and of the MtvR transcript (right panel) in cells of the WT or Δ*hfq* mutant, expressing MtvR (+pMtvR). (C) Levels of the *hfq_Pa_* mRNA and the MtvR transcript in cells of the *P. aeruginosa* WT or WT with the *hfq* gene silenced (*hfq* ^sil^), expressing (+pMtvR) or not MtvR. (D) Analysis of the stability of the *P. aeruginosa hfq_Pa_* mRNA and of the MtvR transcript, in cells of the *P. aeruginosa* strains WT or the WT with the *hfq* gene silenced (*hfq^sil^*), expressing (+pMtvR) or not MtvR. The levels of the 5S rRNA were used as loading control.

We have investigated if MtvR affects *hfq* expression in *E. coli* and *P. aeruginosa.* For this purpose, we transformed *E. coli* and *P. aeruginosa* WT strains with plasmid pCGR12, which allows MtvR expression. Results in Fig. 1A and 1C confirm that the sRNA is expressed, respectively, in *E. coli* and *P. aeruginosa*. The ectopic expression of MtvR for 2, 8 or 24 h led, respectively, to a reduction of 1.4±0.09, 1.29±0.08 or 1.21±0.03 fold of the *hfq*_Ec_ mRNA levels (Fig. 1A), and to a reduction of the *hfq*_Pa_ mRNA levels of 2.60±0.07, 2.30±0.13 or 1.8±0.3 fold (Fig. 1C). Expression of MtvR also affected the stability of the *E. coli* and *P. aeruginosa hfq* mRNAs, leading to the reduction of the *hfq*_Ec_ mRNA half-life time (t_1/2_) from 13.4±1.0 to 8.6±0.5 min (Fig. 1B), and of the *hfq*_Pa_ mRNA t_1/2_ from 18.6±1 to 9.9±0.4 min (Fig. 1D). Unlike the single band corresponding to *hfq*_Ec_ or *hfq*_Pa_ mRNAs observed for the respective controls, the expression of MtvR resulted in the detection of two bands corresponding to *hfq*_Ec_ or *hfq*_Pa_ mRNA decay products, suggesting that the processing of both mRNAs in the presence of MtvR involves pathways distinct from those used by the *E. coli* or *P. aeruginosa* WT strains. Interestingly, our results also indicate that MtvR requires the Hfq proteins from *E. coli* or *P. aeruginosa* for stability, as suggested by the decrease of the sRNA t_1/2 _from 17.3±0.7 min in the *E. coli* WT strain to 8.5±0.4 min in the *E. coli hfq* mutant (Fig. 1B), and from 5.2±0.7 min in the *P. aeruginosa* WT strain to 2.6±0.4 min in the *P. aeruginosa* strain with the *hfq*_Pa_ gene silenced (Fig. 1D). Together, these results indicate that MtvR is stabilized by Hfq_Ec_ and Hfq_Pa_, while the mRNAs *hfq*_Ec_ and *hfq*_Pa_ are targets of this sRNA.

### The MtvR sRNA Binds to the 5′ Leader Region of the *hfq* mRNA

Based on both experimental and bioinformatics analyses indicating that *hfq*_Ec_ mRNA is a target of MtvR, we have conducted further experiments to gain additional insights into the molecular details of the observed negative regulatory effects exerted by MtvR on the *hfq*_Ec_ mRNA.

**Figure 2:**
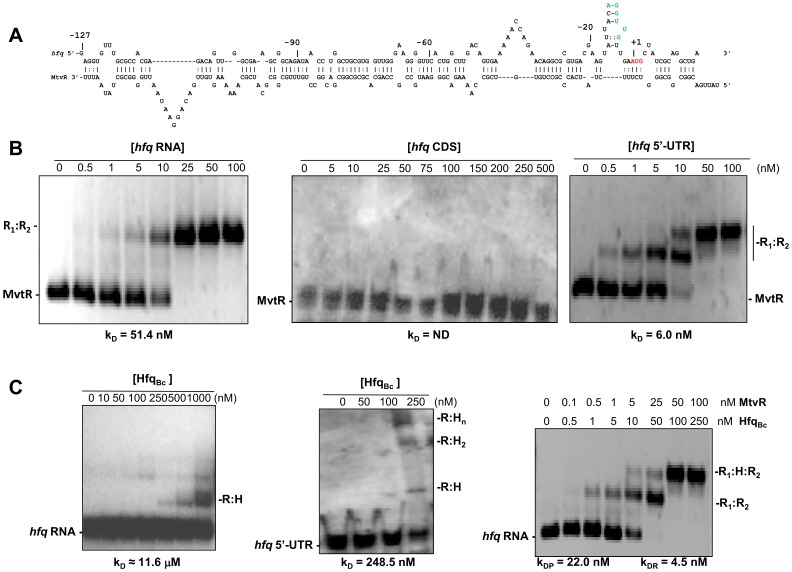
MtvR regulates *hfq*_Ec_ expression through interaction with its 5′-leader region. (A) Schematic representation of the nucleotide interaction between the 5′-UTR of *hfq*_Ec_ and the MtvR sRNA, highlighting in green and red lettering, respectively, the RBS and the AUG translation start site in the *hfq*_Ec_ 5′-UTR. (B) EMSA experiments using 25 nM of biotin-labeled MtvR transcript and the indicated concentrations of: left panel, the *hfq*_Ec_ full mRNA; center panel, the *hfq*_Ec_ coding region (CDS); right panel, the 5′-UTR of *hfq*_Ec_. C) EMSA experiments performed to assess the ability of Hfq_Bc_ to bind to: left panel, 25 nM of the biotin-labeled *hfq*_Ec_ full mRNA; center panel, 25 nM of the biotin-labeled *hfq*_Ec_ 5′-UTR. The ability of mixtures containing Hfq_Bc_ together with the tested concentrations of the MtvR transcript to bind to 25 nM of the biotin-labeled *hfq*_Ec_ full mRNA is shown in the right panel. H_(n)_, Hfq_Bc(n)_; R_1_, MtvR; R_2_, *hfq*_Ec_ RNA; k_DP_, affinity constant for the protein; k_DR_, affinity constant for the RNA species.

The interaction between MtvR and the *hfq*_Ec_ 5′ leader region was investigated using the RNAHybrid software, which predicted a strong interaction between the two RNA molecules, suggesting that these two RNAs are able to form extended RNA duplexes, occluding the Ribosome Binding Site (green letters in Fig. 2A) and the start codon (Red letters in Fig. 2A).

This RNA-RNA interaction was experimentally confirmed by EMSA experiments. For this purpose, MtvR was transcribed *in vitro* and biotin-labeled, and the *hfq*_Ec_ RNA was transcribed from a T7 promoter using as template plasmid pCGR36, yielding the complete transcriptional unit of *hfq*_Ec_, composed of 475 nt corresponding to the 5′ leader region of *hfq*_Ec_, and 306 nt corresponding to the *hfq*_Ec_ coding sequence (CDS). Results presented in Fig. 2B (left panel) show that MtvR binds to the *hfq_Ec_* RNA, with an apparent K_D_ of 51.4 nM. Similar band-shift assays performed with the *hfq_Ec_* CDS instead of the full RNA, revealed that MtvR requires the *hfq_Ec_* 5′-UTR for binding (Fig. 2B, center panel), since no MtvR displacement could be detected, at least for the concentrations used of the *hfq*_Ec_ CDS. An additional experiment was performed using the sRNA together with the 5′-UTR of *hfq_Ec_*. Results shown in Fig. 2B (right panel) indicate that MtvR binds to the 5′-UTR of *hfq_Ec_*, with an apparent K_D_ of 6.0 nM.

The *E. coli* Hfq protein was previously shown to be auto-regulated, through the interaction of the protein with two distinct binding sites within the 5′ leader of the mRNA, resulting in the inhibition of the formation of the translation initiation complex [25]. Since the action of sRNAs is often mediated by the Hfq RNA chaperone and the Hfq protein is involved in auto-regulation in *E. coli*, apparently without the requirement for sRNAs, we have conducted EMSA experiments with the *hfq_Ec_* RNA (composed of the 5′-UTR and the coding sequence), or the *hfq_Ec_* 5′-UTR together with the Hfq of *B. cenocepacia* J2315 (Hfq_Bc_, which lacks amino acid residues beyond position 79). Results obtained indicate that Hfq_Bc_ can form complexes with the *hfq_Ec_* RNA only in relatively high concentrations (500 nM or higher) (Fig. 2C, left panel), with an apparent K_D_ of 11.6 µM. The Hfq_Bc_ protein also needs to be present in concentrations as high as 250 nM in order to bind to the *hfq_Ec_* 5′-UTR RNA (Fig. 2C, center panel). Interestingly, in the presence of the MtvR sRNA, the Hfq_Bc_ protein is able to bind to the two RNAs at a concentration 500-fold lower compared to the *hfq_Ec_* RNA alone (Fig. 2C, left panel).

These results suggest that MtvR together with Hfq_Bc_ can synergistically bind to the *hfq*_Ec_ mRNA, more efficiently than Hfq_Bc_ alone.

### MtvR Expression Affects *E. coli* Hfq Translation

Our data strongly suggests that MtvR might also impact the Hfq_Ec_ levels. To further investigate the impact of MtvR on the levels of Hfq_Ec_, we have transformed the *E. coli* WT strain and the strain expressing MtvR with plasmid pCGR34, which allows expression of Hfq_Ec_-His from its own promoter. This was performed since the polyclonal anti-Hfq IgG that was previously raised (unpublished data) is specific for the Hfq_Bc_, and no signals on Western blots could be detected for the Hfq_Ec_ using this antibody (data not shown).

**Figure 3:**
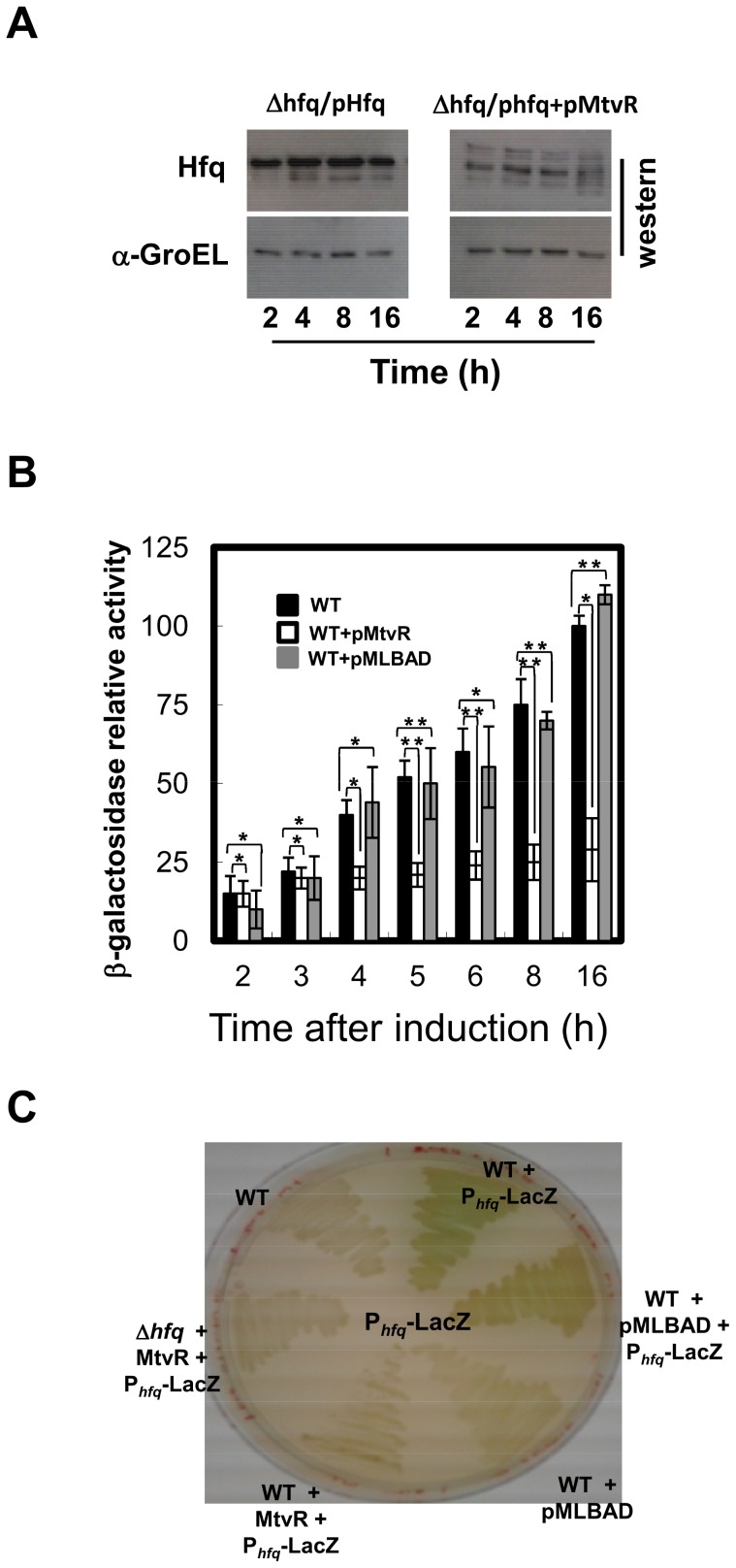
MtvR affects *E. coli* Hfq translation. (A) Western blot analysis of the levels of the 6×His-tagged Hfq_Ec_ protein in cells of the *E. coli* Δ*hfq* mutant expressing only the tagged protein (left panel, Δ*hfq*/p*hfq*), or also expressing the MtvR transcript (Right panel, Δ*hfq*/p*hfq*+pMtvR). The GroEL levels were used as loading control. (B) β-galactosidase relative activity in cells of the *E. coli* strains WT (black bars), WT expressing MtvR (+pMtvR, white bars), or WT with the empty vector (pMLBAD, grey bars), harboring the *hfq*_Ec_ 5′-UTR-LacZ fusion (p*_hfq_*-LacZ). C) β-galactosidase activity of the indicated *E. coli* strains, grown in solid LB media, supplemented with X-Gal and 0.1% L-arabinose. The WT strain transformed with pMLBAD was used as control.

Results from Western blot analysis showed that MtvR expression reduced *hfq*_Ec_ translation by 1.87-, 2.09-, 2.30- or 2.77-fold, at 2, 4, 8 or 16 h, respectively (Fig. 3A).

The *in vivo* assessment of MtvR interaction with the 5′ leader region of *hfq*_Ec_ was achieved by measuring the β-galactosidase activity of the 5′-UTR-LacZ fusion. With this purpose, *E. coli* derivatives expressing MtvR were transformed with plasmid pCGR35. The WT strain was transformed with pMLBAD and used as control. Results obtained (Fig. 3B and 3C) show that the lowest values of β-galactosidase activity were registered when MtvR was expressed. The inability of MtvR to induce a complete translational blocking might derive from its dependence on a RNA chaperone for increased stability. These results are consistent with a negative regulatory action exerted by MtvR on *hfq*_Ec_ mRNA, occurring by binding to its 5′ leader region, which most probably prevents an efficient translation of the messenger RNA, by coupling enhanced mRNA decay with impaired ribosome loading.

### MtvR Expression Affects *E. coli* and *P. aeruginosa* Growth Abilities

**Figure 4:**
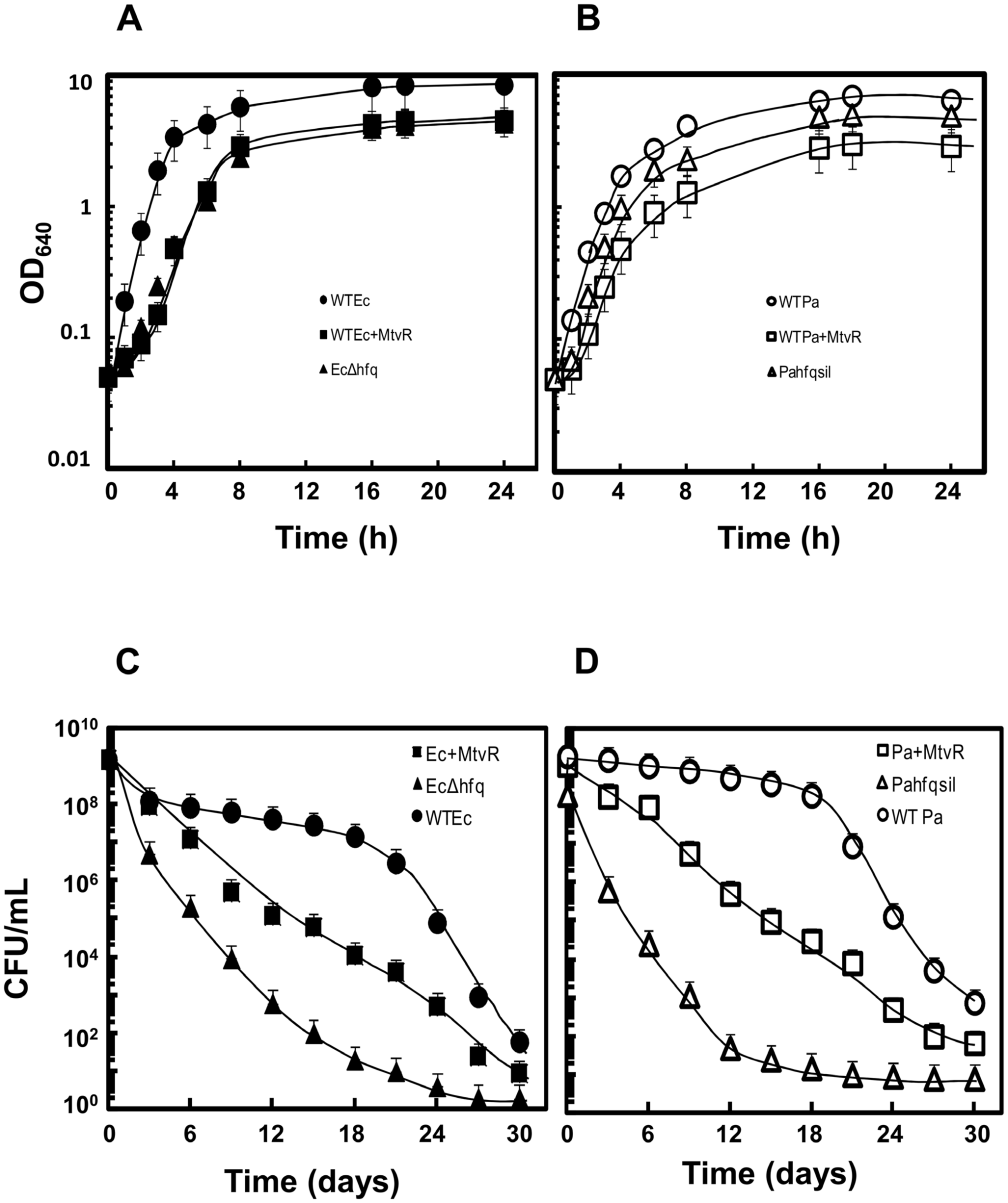
MtvR expression affects *E. coli* and *P. aeruginosa* growth kinetics and survival to prolonged nutrient deprivation. Growth curves in liquid LB medium supplemented with 0.1% L-arabinose (A, B) and survival in M9 minimal medium supplemented with 0.1% L-arabinose, at 37°C for 30 days (C, D), of (A, C) *E. coli* strains WT (circles), WT expressing MtvR (squares) or the Δ*hfq*_Ec_ mutant (triangles), or (B,D) *P. aeruginosa* strains WT (circles), WT expressing MtvR (squares) or the WT strain with the *hfq*_Pa_ gene silenced (triangles).

Data presented so far indicates that MtvR is able to regulate *hfq*_EC_ and *hfq*_Pa_, and, at least, Hfq_Ec_. This led us to investigate the roles of MtvR on the growth abilities of *E. coli* and *P. aeruginosa*. The specific growth rate and biomass yield reached after 24 h of batch growth of the *E. coli* expressing MtvR were reduced to approximately the same level as that of the Δ*hfq* mutant strain (Fig. 4A). In the case of *P. aeruginosa*, the expression of MtvR reduced the specific growth rate and biomass yield reached after 24 h of batch growth to levels even lower than those observed for the strain with the *hfq*_Pa_ silenced (Fig. 4B). When challenged with long-term carbon starvation, *E. coli* and *P. aeruginosa* strains expressing MtvR exhibited a reduction of their survival ability more pronounced than the reduction observed for the *E. coli* Δ*hfq* mutant and the *P. aeruginosa* with the *hfq* gene silenced (*hfq* ^sil^), respectively.

Since the growth rate and biomass yield or resistance to nutrient deprivation registered for the WT strains of *E. coli* or *P. aeruginosa* and the respective transformants harboring pMLBAD were similar, these results were not included in Fig. 4 to keep it simpler.

### MtvR Affects the Biofilm Formation Ability of *E. coli* and *P.* *aeruginosa*

**Figure 5:**
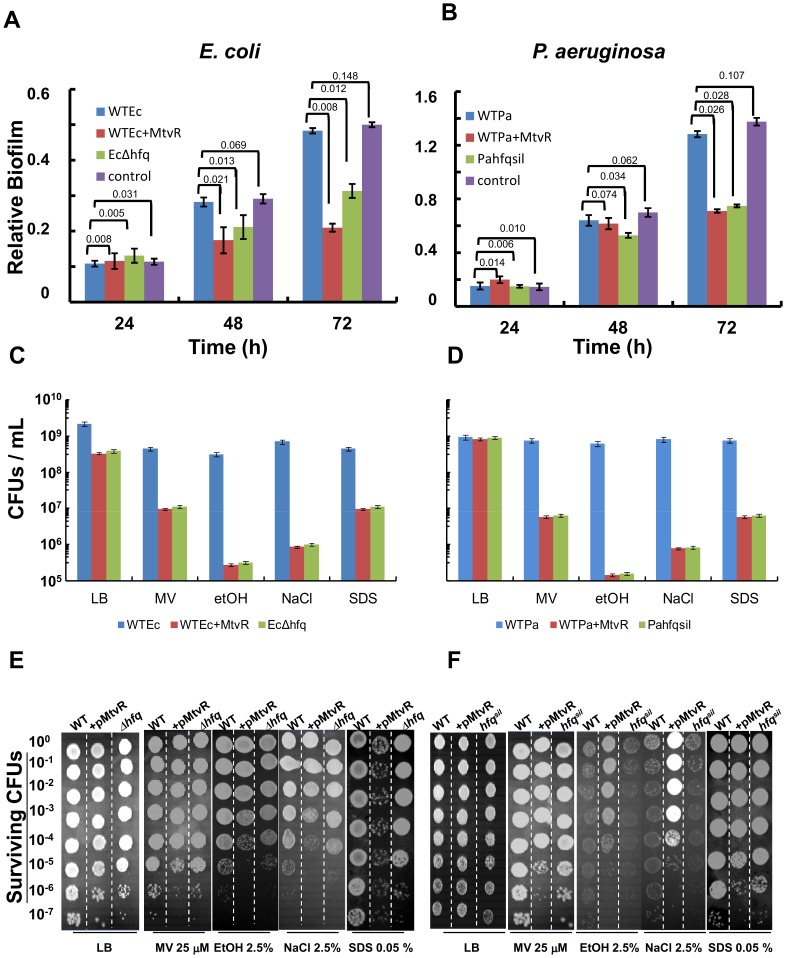
MtvR expression in *E. coli* and *P. aeruginosa* reduces biofilm formation ability and increases susceptibility to stresses. Relative biofilm formation ability (panels A, B) and susceptibility to the stress imposed by growth on the surface of LB solid medium supplemented or not (LB) with the indicated concentrations of methyl viologen (MV), ethanol (etOH), NaCl or SDS (SDS) (panels C,D), of strains of (panels A, C) *E. coli* WT (WTEc), WT expressing MtvR (WTEc+MtvR), or the *Δhfq*_Ec_ mutant (Ec*Δhfq*), and (panels B, D) *P. aeruginosa* WT (WTPa), WT expressing MtvR (WTPa+MtvR), or the WT strain with the *hfq*_Pa_ gene silenced (Pahfqsil). Relative biofilm formation was estimated by dividing the total amount of biofilm formed by the total amount of biomass (see Materials and Methods section). Panels E and F show photographs illustrative of results from a single representative susceptibility experiment with the *E. coli* (panel E) and *P. aeruginosa* (panel F) strains WT (WT), WT expressing MtvR (+MtvR), and the *E. coli* Δ*hfq*_Ec_ mutant (Δ*hfq*), or the *P. aeruginosa* WT with the *hfq*_Pa_ gene silenced (*hfq* ^sil^). Susceptibilities were assessed by spot inoculation of serially diluted bacterial suspensions with an initial OD640 of 1.0. Error bars represent standard deviation of the means. Numbers above bars in panels (A) and (B) are the estimated P-values.

In a previous study MtvR was shown to play a role on biofilm formation ability in Bcc bacteria [9]. Therefore, we decided to investigate the effect of expressing MtvR on the biofilm formation ability of *E. coli* and *P. aeruginosa*. Since for the different strains under study differences in the biomass yield were registered, results were expressed as relative biofilm amount, i.e., the ratio of biofilm amount estimated using the crystal violet and the biomass concentration assessed by the OD_640_ of cultures. Results obtained are presented in Figs. 5A and 5B and indicate that MtvR expression affected negatively the ability of both *E. coli* and *P. aeruginosa* to form biofilms *in vitro*, particularly evident at 72 h. The observed effects might result from the down-regulation of *hfq*_Ec_ and *hfq*_Pa_, since the strains *E. coli* Δ*hfq* and *P. aeruginosa hfq* ^sil^ formed relative biofilm amounts comparable to those formed by the respective WT strains expressing MtvR.

### *E. coli* and *P. aeruginosa* Exhibit Enhanced Susceptibility to Stresses When Expressing MtvR

The effects of MtvR expression on the susceptibility of *E. coli* and *P. aeruginosa* strains to oxidative, osmotic and membrane stresses were investigated by spot-inoculation of bacterial culture aliquots on LB solid media supplemented with methyl viologen, NaCl, ethanol or SDS. Illustrative photographs of a set of results obtained for *E. coli* and *P. aeruginosa* are shown, respectively, in Figs. 5E and 5F. Methyl viologen is a charged quaternary ammonium compound that generates reactive oxygen species under aerobic conditions [26], used to generate oxidative stress conditions. Ethanol and NaCl were used to increase the medium osmolarity, while SDS was used as a membrane integrity-perturbing agent. These stressors were chosen to mimic the environmental conditions oxidative stress, high osmolarity, and extracytoplasmic stress, conditions that are faced, for instance, when *P. aeruginosa* colonizes/infects the cystic fibrosis lung [10].

Results presented in Fig. 5C show that when compared to the WT strain, the numbers of total CFUs of the *E. coli* strains expressing MtvR or the Δ*hfq*_Ec_ mutant, exposed to methyl viologen or SDS, were reduced by more than 1 log. This reduction was higher, 2 to 3 logs, for cells of the strains expressing MtvR or Δ*hfq*_Ec_ when exposed to stressors NaCl or ethanol, respectively. Under non-stress conditions, about one log reduction in the total CFU were registered for the *E. coli* strains expressing MtvR and the mutant Δ*hfq*_Ec_ when compared to the WT strain (Fig. 5C). It is worth to note that the reductions in total CFUs registered for the *E. coli* strains expressing MtvR or the Δ*hfq* mutant were quite similar, suggesting that the observed increased susceptibility to the tested stressors might result from the down-regulation of *hfq*_Ec_.

In the case of *P. aeruginosa*, no differences on the total CFUs were registered for the strains WT, WT expressing MtvR, and the WT with the *hfq*_Pa_ gene silenced under non-stress conditions (Fig. 5D). However, when compared to the *P. aeruginosa* WT strain, approximately 2, 3, or 4-log reduction in the total CFU were registered for the strains *P. aeruginosa* expressing MtvR and *P aeruginosa hfq* ^sil^ when exposed, respectively, to methyl viologen or SDS, NaCl, or ethanol (Fig. 5D). Since no significant differences in the total CFUs were observed for the *E. coli* strains WT and WT transformed with pMLBAD, and *P. aeruginosa* WT and *P. aeruginosa* transformed with pMLBAD, only the results obtained for the *E. coli* and *P. aeruginosa* WT strains are shown, respectively, in Figs. 5C to 5F.

### MtvR Expression Enhances Antibiotic Susceptibility in *E.* *coli* and *P. aeruginosa*

The sRNA MtvR was recently shown to play a role on resistance to antibiotics in Bcc [9]. Therefore, we investigated the effects of MtvR expression on the *E. coli* and *P. aeruginosa* susceptibility to the antibiotics chloramphenicol, ciprofloxacin, tetracycline, tobramycin, gentamycin and ampicillin. Results presented in Table 3 show that MtvR expression reduced *E. coli* MIC values by 8-fold for tetracycline, 4-fold for chloramphenicol and ciprofloxacin, and 2-fold for tobramycin, gentamycin and ampicillin. The observed increased susceptibility to these antibiotics in the Δ*hfq*_Ec_ mutant strain was identical to the observed due to MtvR expression, except for chloramphenicol and tetracycline. For these two antibiotics, the susceptibility only increased by 2- and 4-fold, respectively. The effect of MtvR expression in the Δ*hfq*_Ec_ led to an even more drastic effect in susceptibility, especially to tobramycin and gentamycin, with MIC values lowering 16- and 128-fold, respectively (Table 3).

**Table 3:** Antibiotic susceptibility of *E. coli* WT and derivative strains.

Strain	CHL	CIP	TET	NM	GNT	AMP
*E. coli* WT	4	1	2	8	16	4
*E. coli* Δ*hfq*	2*	0.25	0.25	2	8	2
*E. coli* WT/p*hfq*	16	8	32	32	128	64
*E. coli* WT/p*hfq*+MtvR	2	0.25	0.5	2	1	0.5
*E. coli* WT/pMLBAD	4	1	2	8	16	4
*E. coli* WT/MtvR	1	0.125	0.125	4	8	2
*E. coli* Δ*hfq*/pMLBAD	64*	≤0.125	0.125	1	8	2
*E. coli* Δ*hfq*/p*hfq*	>512*	1	2	8	16	4
*E. coli* Δ*hfq*/MtvR	8*	≤0.125	≤0.125	0.5	0.125	1
*E. coli* Δ*hfq*/p*hfq*+MtvR	64*	0.125	0.125	4	8	2

CHL, chloramphenicol; CIP, ciprofloxacin; TET, tetracycline; NM, tobramycin; GNT, gentamycin; AMP, ampicillin. Numbers with asterisks represent strains that have a chromosomal chloramphenicol resistance cassette.

We have also investigated the role of MtvR on the *P. aeruginosa* susceptibility to chloramphenicol, ciprofloxacin, tetracycline, tobramycin, gentamycin and ampicillin. Results in Table 4 indicate that the antibiotic susceptibility to the tested antibiotics increased in cells expressing MtvR. MtvR expression induced a 4-fold reduction in most of the MIC values, with the exception of gentamycin, for which a MIC value 2-fold lower than the WT strain was registered (Table 4). In the strain with the *hfq*_Pa_ gene silenced, the antibiotic susceptibility was also affected, although to a lesser extent. The MIC values for chloramphenicol and ampicillin remained unchanged in the strain with the *hfq* silenced (Table 4). A more drastic effect on antibiotic susceptibility was observed in the strain with the *hfq* gene silenced and expressing MtvR. In fact, an impressive 64-fold reduction in the MIC value for chloramphenicol, 32-fold for tobramycin and gentamycin, and 16-fold for ampicillin were registered for this strain (Table 4).

**Table 4:** Antibiotic susceptibility of *P. aeruginosa* WT and derivative strains.

Strain	CHL	CIP	TET	NM	GNT	AMP
*P. aeruginosa* WT	64	1	16	16	128	>512
*P. aeruginosa hfq^sil^*	64	0.5	8	4	64	512
*P. aeruginosa* WT/p*hfq*	128	1	64	128	256	>512
*P. aeruginosa* WT/p*hfq*+MtvR	16	0.125	2	4	16	64
*P. aeruginosa* WT/pMLBAD	64	1	16	16	128	>512
*P. aeruginosa* WT/MtvR	16	0.125	2	4	16	64
*P. aeruginosa hfq^sil^*/MtvR	1	0.125	1	0.5	8	32

CHL, chloramphenicol; CIP, ciprofloxacin; TET, tetracycline; NM, tobramycin; GNT, gentamycin; AMP, ampicillin.

### MtvR has Additional Putative Targets in *E. coli*

Some of the phenotypes here reported for the *E. coli* strain expressing MtvR differ from those observed for the *E. coli* Δ*hfq*_Ec_ mutant, suggesting that MtvR might regulate additional mRNAs in this bacterium. Therefore, we have used the programs TargetRNA [21], RNAPredator [22] and sRNATarget [20] to predict possible MtvR additional mRNA targets within the genome of *E. coli* K12 strain MG1655. A total of 11 (Table S1 in File S1) and 3 (Table S2 in File S1) putative mRNA targets were predicted by TargetRNA and RNAPredator, respectively, assuming only putative hybridizations in the −20 to +20 nt region around the start codon. Using the sRNATarget with the same restrictions, and excluding genes of unknown functions, 52 distinct mRNA targets were predicted (Table S3 in File S1). Interestingly, several targets were predicted to interact with MtvR in more than one region (Table S4 in File S1).

**Figure 6:**
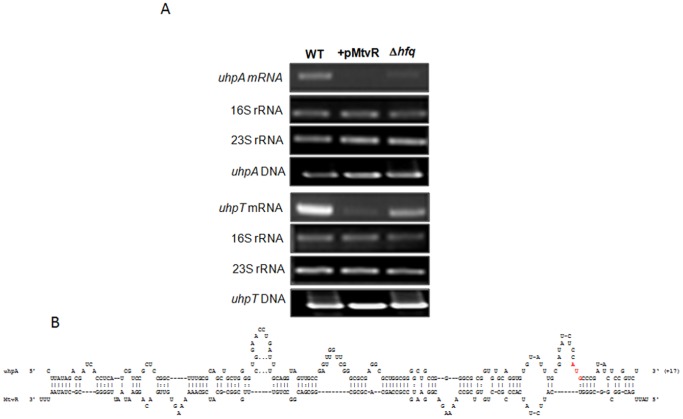
The MtvR sRNA also targets *uhpA* gene in *E. coli*. (A) Reverse-transcription analysis of the effect of MtvR on the mRNA levels of *uhpA*. Total RNA was obtained from late-exponentially growing cells of the *E. coli* strains WT and WT expressing MtvR (+pMtvR), or the Δ*hfq* mutant (Δ*hfq*). Reverse-transcription experiments were also performed for *uhpT*, induced by UhpA. The 16S and 23S rRNA bands were used as loading controls. PCR experiments were also performed using DNA, for reference. Images shown are representative of 3 independent experiments. B) Schematic representation of the nucleotide interaction between the 5′-UTR of *uhpA* and MtvR, highlighting in red lettering the AUG translation start site in the 5′-UTR of *uhpA*.

The gene *uhpA* of *E. coli* was the only common target of MtvR predicted by TargetRNA and sRNATarget. The *uhpA* encodes the response regulator of a two-component regulatory system where UhpB is a histidine kinase that controls the synthesis of the sugar-phosphate transporter UhpT [27]. These findings prompted us to investigate the effects of MtvR expression on the transcript levels corresponding to the *uhpA* mRNA in *E. coli* using RT-PCR. We have also investigated the levels of the *uhpT* mRNA, which is regulated by the UhpA-UhpB two-component regulatory system [27]. Results presented in Fig. 5 show that MtvR expression led to undetectable *uhpA*-derived cDNA, opposed to the observed for the WT strain (Fig. 6A). However, cDNA corresponding to *uhpA* mRNA could be detected in the Δ*hfq*_Ec_ mutant strain, although with a reduced intensity relative to the WT strain. These observations suggest that Hfq might be involved in *uhpA* regulation.

The levels of cDNA corresponding to *uhpT* mRNA were highly reduced in *E. coli* cells expressing MtvR when compared to those observed for the WT strain (Fig. 6A). This observation is consistent with the requirement of UhpA (in its phosphorylated form) for the transcriptional activation of *uhpT* [27].

Since the levels of cDNA corresponding to *uhpT* mRNA were higher in the Δ*hfq*_Ec_ mutant than in the strain expressing MtvR, we concluded that Hfq_Ec_ is unlikely to be involved in the direct regulation of *uhpT* mRNA. The regulation exerted by MtvR on *uhpA* mRNA might involve partial base-pairing of the two molecules, predicted to occur within the 5′ region of *uhpA* mRNA, leading to the formation of a RNA duplex (with a predicted energy of −117.0 kcal/mol) that occludes part of the RBS and start codon (Fig. 6B).

We also have used the TargetRNA [21], the RNAPredator [22] and the sRNATarget [20] programs to predict possible MtvR mRNA targets within the genome of *P. aeruginosa* UCBPP-PA14 (Tables S5 and S6 in File S1). No common targets were predicted.

## Discussion

The sRNA MtvR was recently identified as a *trans*-encoded sRNA that occurs exclusively among members of the *Burkholderia* genus [9]. In these bacteria, MtvR acts as a global regulatory RNA, and strains with the sRNA silenced or overexpressed exhibited pleiotropic phenotypes related to growth and survival when challenged with stress, motility, biofilm formation, resistance to antibiotics and virulence [9]. In addition, MtvR was shown to regulate the levels of at least 17 mRNA targets in the cystic fibrosis isolate *B. cenocepacia* J2315 [9]. Results presented in this work also show that, at least in *E. coli*, MtvR targets other genes besides *hfq*_Ec_, as is the case of *uhpT*.

Several *trans*-encoded sRNAs have been described as regulating multiple targets, most probably due to the limited complementarities shared with their mRNA targets [6]. This limited base-pairing is thought to justify the need of most of the *trans*-encoded sRNAs to bind to the RNA chaperone Hfq to effectively interact with their targets [6].

Despite the absence of homologues to MtvR in *E. coli* or *P. aeruginosa*, here we present evidence that this sRNA is able to regulate the levels of the *hfq* mRNA in both species.

The Hfq protein of *Burkholderia* is 83% identical to the *E. coli* [14]. The *E. coli* Hfq is composed of 102 amino acid residues, while the *P. aeruginosa* and *B. cenocepacia* Hfq proteins are composed of 82 and 79 amino acid residues, respectively. All 3 proteins contain the conserved Sm1 and Sm2 motifs, and have a secondary structure compose by a N-terminal α-helix, followed by 5 β-strands. The amino acid residues 8–68 correspond to the conserved core of the 3 proteins.

Earlier studies on the *E. coli* Hfq have shown that the protein relative abundance is growth-phase dependent [28], being *hfq*_Ec_ transcription regulated by multiple mechanisms [29]. More recently, Vecerek *et al*. (2005) presented evidence indicating that in *E. coli* the synthesis of Hfq_Ec_ is auto-regulated at the translational level [25]. These authors have shown that Hfq_Ec_ binds to the two Hfq_Ec_-binding sites in the 5′-UTR region of *hfq*_Ec_ mRNA, inhibiting the formation of the translation initiation complex [30], in an interaction that involves the C-terminal region of the protein. This interaction occurs *in vitro* at protein concentrations ranging 50–200 nM [30]. In the case of Bcc bacteria, the Hfq_Bc_ protein lacks the C-terminal region [12] suggesting a lack of auto-regulation. In fact, the *B. cenocepacia hfq*_Bc_ mRNA was recently shown to be regulated through a mechanism involving sequestration of the RBS by MtvR, leading to accelerated mRNA decay and reduced protein translation [9].

Interestingly, the 5′-UTR region of the Bcc *hfq*_Bc_ is 55% identical to the *E. coli hfq*_Ec_ 5′-UTR (data not shown). Our results show that the *B. cenocepacia* shorter protein is also able to bind to the *E. coli hfq*_Ec_ 5′ leader region, but with a ∼100-fold lower affinity. We also show that the sRNA can act synergistically with Hfq_Bc_, increasing its affinity to *hfq*_Ec_ mRNA by more than 500-fold.

A few examples of functional analysis of sRNAs on heterologous systems have been reported. For instance, AbdelRahman *et al.* (2010) have used a heterologous co-expression system to demonstrate that the *Chlamydia trachomatis* non-coding RNA CTIG270 regulates the expression of FtsI by inducing *ftsI* mRNA degradation [31].

The data presented here show that despite being absent in *E. coli*, MtvR regulates at least, the levels of the *hfq*, *uhpA* and *uhpT* mRNAs. In *B. cenocepacia* J2315, MtvR affects the mRNA levels of at least 17 genes, among 309 predicted targets [9]. It is therefore possible that MtvR also interacts with other mRNAs, as suggested by the observed phenotypes of cells expressing the sRNA.

Perhaps the most interesting phenotypes observed due to MtvR expression in *E. coli* and *P. aeruginosa* are those related to increased antibiotic susceptibility. Bacterial resistance to antibiotics is a problem of increasingly concern, since data from the Centers for Disease Control and Prevention evidence a rapidly increasing rate of infections due to fluoroquinolone-resistant *P. aeruginosa* [32].

A recent study revealed that the involvement of MtvR in the regulation of an *hfq*-like gene also impacted the bacterium resistance to several antibiotics, leading to a phenotype conversion from resistant to susceptible [9]. A study by Yamada *et al.* (2010) revealed that mutations in the *hfq* gene from *E. coli* resulted in susceptibility to acriflavine, benzalkonium, cefamandole, chloramphenicol, crystal violet, nalidixic acid, novobiocin, oxacillin and rhodamine 6G [33]. In addition, Moon & Gottesman (2009) reported on the requirement of Hfq for resistance to polymyxin B through a mechanism involving the sRNA MgrR [34]. In *Stenotrophomonas maltophilia*, Hfq was also shown to play a role in resistance to tobramycin and amikacin, most likely due to the regulation of efflux pumps [35].

Anti-sense acting oligonucleotides are being used as components of peptide-morpholino oligonucleotide conjugates (PMO) that can act as bactericidal agents. For instance, a PMO targeting the highly conserved region of the *E. coli gyrA* was recently shown to effectively inactivate several species of Gram-positive and Gram-negative bacteria, when used in the micro-molar range [36].

Results presented in this work show that when expressing MtvR, the MIC values for the studied antibiotics were reduced by 2 to 128 fold in *E. coli* and *P. aeruginosa*, pointing out this sRNA as an interesting molecule, with potential to be exploited as an adjuvant in antimicrobial therapies. In fact, multi-target sRNAs, like MtvR, are potential candidates for the development of PMOs that can be used as antimicrobials, or in combination with already available antibiotics, to fight infections by multi-resistant bacteria, as is the case of *P. aeruginosa*.

## Supporting Information

File S1 Supporting Information File combining Supplementary Methods, Supplementary Results, Table S1 (Putative MtvR targets in the genome of *E. coli* predicted by TargetRNA), Table S2 (Putative MtvR targets in the genome of *E. coli* predicted by RNAPredator), Table S3 (Putative MtvR targets in the genome of *E. coli* predicted by sRNATarget), Table S4 (Regions with homology to within the genome of *E. coli*), Table S5 (Putative MtvR targets in the genome of *P. aeruginosa* UCBPP-PA14 predicted by TargetRNA), and Table S6 (Putative MtvR targets in the genome of *P. aeruginosa* UCBPP-PA14 predicted by RNAPredator), and Supplementary References.
(PDF)
