# Correction: Regulation of Hfq mRNA and Protein Levels in *Escherichia coli* and *Pseudomonas aeruginosa* by the *Burkholderia cenocepacia* MtvR sRNA

**DOI:** 10.1371/journal.pone.0116066

**Published:** 2014-12-16

**Authors:** The PLOS ONE Staff

Figure 5E is incorrect. Please view the corrected Figure 5 here.

**Figure 5:**
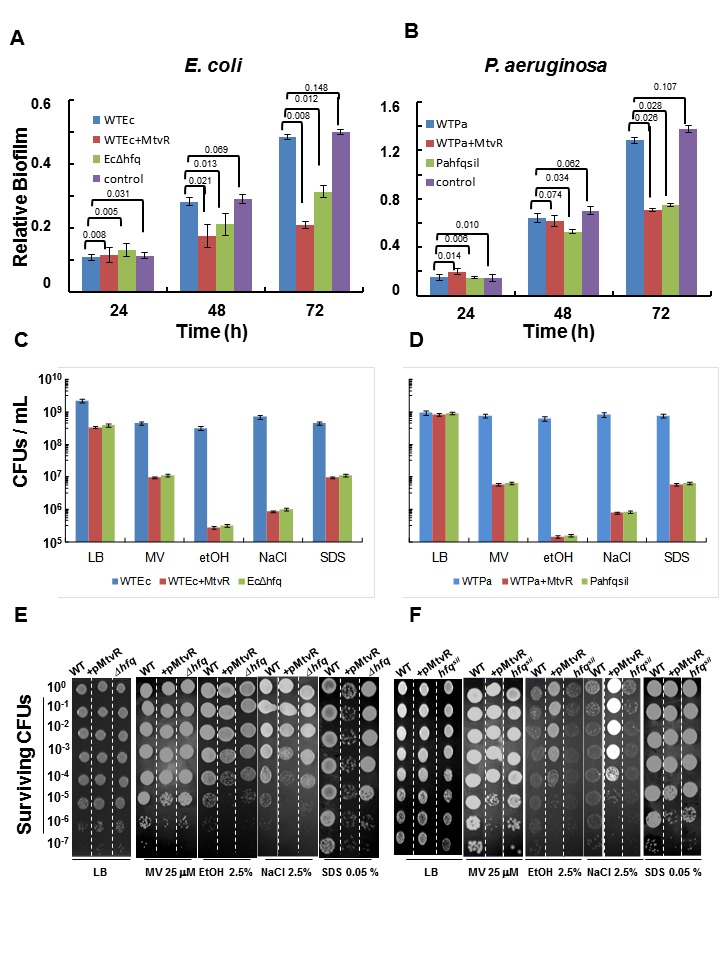
MtvR expression in *E. coli* and *P. aeruginosa *reduces biofilm formation ability and increases susceptibility to stresses. Relative biofilm formation ability (panels A, B) and susceptibility to the stress imposed by growth on the surface of LB solid medium supplemented or not (LB) with the indicated concentrations of methyl viologen (MV), ethanol (etOH), NaCl or SDS (SDS) (panels C,D), of strains of (panels A, C) *E. coli* WT (WTEc), WT expressing MtvR (WTEc+MtvR), or the *Δhfq*_Ec_ mutant (Ec*Δhfq*), and (panels B, D) *P. aeruginosa* WT (WTPa), WT expressing MtvR (WTPa+MtvR), or the WT strain with the *hfq*_Pa_ gene silenced (Pahfqsil). Relative biofilm formation was estimated by dividing the total amount of biofilm formed by the total amount of biomass (see Materials and Methods section). Panels E and F show photographs illustrative of results from a single representative susceptibility experiment with the *E. coli* (panel E) and *P. aeruginosa* (panel F) strains WT (WT), WT expressing MtvR (+MtvR), and the *E. coli* Δ*hfq*_Ec_ mutant (Δ*hfq*), or the*P. aeruginosa* WT with the *hfq*_Pa_ gene silenced (*hfq* ^sil^). Susceptibilities were assessed by spot inoculation of serially diluted bacterial suspensions with an initial OD640 of 1.0. Error bars represent standard deviation of the means. Numbers above bars in panels (A) and (B) are the estimated P-values.
