# Supplementary material for: Regulation of Hfq mRNA and Protein Levels in Escherichia coli and Pseudomonas aeruginosa by the Burkholderia cenocepacia MtvR sRNA
Source: PLoS One. 2014 Jun 5;9(6):e98813. doi: 10.1371/journal.pone.0098813 (PMC4046987; doi:10.1371/journal.pone.0098813)
Supplement: File S1 — Supporting Information File combining Supplementary Methods, Supplementary Results, Table S1 (Putative MtvR targets in the genome of E. coli predicted by TargetRNA), Table S2 (Putative MtvR targets in the genome of E. coli predicted by RNAPredator), Table S3 (Putative MtvR targets in the genome of E. coli predicted by sRNATarget), Table S4 (Regions with homology to within the genome of E. coli), Table S5 (Putative MtvR targets in the genome of P. aeruginosa UCBPP-PA14 predicted by TargetRNA), and Table S6 (Putative MtvR targets in the genome of P. aeruginosa UCBPP-PA14 predicted by RNAPredator), and Supplementary References. (PDF) [file pone.0098813.s001.pdf]

Supplementary information

Regulation of Hfq mRNA and protein levels in *Escherichia coli* and *Pseudomonas aeruginosa* by the *Burkholderia cenocepacia* MtvR sRNA

Christian G. Ramos, André M. Grilo, Sílvia A. Sousa, Joana R. Feliciano, Paulo J. P. da Costa and Jorge H. Leitão*

Department of Bioengineering and Institute for Biotechnology and Bioengineering, Instituto Superior Técnico, Universidade de Lisboa, Av. Rovisco Pais, Torre Sul, Piso 6, 1049-001, Lisboa, Portugal.

*Corresponding author: Fax: +351 218419199; E-mail: jorgeleitao@tecnico.ulisboa.pt

Supplementary Methods

Bioinformatic analysis

BLAST searches were performed using the Integrated Microbial Genomes (IMG) webserver [1], using an E-value ≤ $1e^{-50}$ as cut-off. MtvR putative targets were predicted using the sRNATarget [2], the TargetRNA [3], and the RNAPredator [4] programs, with a cut-off of 1, a minimum seed of 7 nucleotides, an hybridization target region window size of -100 to + 30 around the translation start site.

Suplementary Results

Interaction of MtvR with additional *E. coli* sequences

A Blast search within the genome of *Escherichia coli* strain K-12 sub-strain MG1655, using a low complexity match and a word size of 6 revealed 9 intergenic regions with segments with homology to the *mtvR* sequence. The non-coding region between *lysV* and REPv173 contains the Intergenic Repeat Region (IRR) rtV, and within this region, the sequence 5'-cgcggcgacggcc-3', corresponding to nt 68 to 80 of MtvR. The rlQ, also a IRR found in the non-coding region between *lysQ* and *nadA*, contains exactly the same sequence of MtvR found on rtV. A H-NS binding sequence of *E. coli* was also found in the non-coding region between yciG and trpA, sharing homology with nt 53-64 (5'-aatgccgccagc-3') of MtvR. The remaining 6 sequences had no identifiable features worth mentioning (Table S4).

This 13 nucleotide sequence match found within non-coding regions of the *E. coli* genome, together with its conservation along other enteric might represent an unidentified regulatory region in *E. coli.*

Additional sequences in *P. aeruginosa* PA14 genome with possible interaction with MtvR

Blast analysis using the MtvR encoding sequence to query the genome sequence of *P. aeruginosa* UCBPP-PA14, revealed a 100% match in a 17 nt (5'-GCCGCGCGTGCTGG-3') segment within an intergenic region 232 bp at the 5' side of a probable C4-dicarboxylate transporter and 550 bp at the 3' side of a arginine/ornithine antiporter. A thorough search revealed that this sequence is widely distributed along all organisms, including prokaryotes, eukaryotes and virus. Another search, using the

complete intergenic region, revealed a 38 nt region (5'-AAATGAAGAAGCCCGGCGAACGCCGGGCTTCTTCATTT-3') that is present only in prokaryotes, and in general, with a conservation over 80 %, found in intergenic regions. No conserved regulatory elements could be identified in this sequence. This sequence might represent a new uncharacterized regulatory region.

Table S1: Putative MtvR targets in the genome of *E. coli* predicted by TargetRNA

1 ***hofN*** protein required for the utilization of DNA as a carbon source; fimbrial assembly protein homolog

Energy: -17.32 p-value: 0.000

```
sRNA          22   CUUAC-UGGGCGGC   10
                    :||| |||||||
mRNA (hofN)   -2   UGAUGAACCCGCCA   12
```

---

2 ***malS*** alpha-amylase

Energy: -16.82 p-value: 0.000

```
sRNA          18   CUGGGCGGCGG-CAGU   4
                    ||:||||||| ||:
mRNA (malS)   5    AACUCGCCGCCUGUUU   20
```

---

3 ***yidJ*** predicted sulfatase/phosphatase

Energy: -16.75 p-value: 0.000

```
sRNA          128  CGCGGGGUUAAUAAGGA   112
                    |||||||||| || ||
mRNA (yidJ)   6    ACGCCCCAAUU-UU-CU   20
```

---

4 ***yjbG*** conserved protein

Energy: -15.58 p-value: 0.000

```
sRNA          62   ACCGCCGUA   54
                    |||||||
mRNA (yjbG)   -13  AGGCGGCAA   -5
```

---

5 **nagC** DNA-binding transcriptional dual regulator, repressor of N-acetylglucosamine

Energy: -14.61 p-value: 0.001

```
sRNA          40   GCUGU-GUCCGCCC   28
                   :|||| |||||||
mRNA (nagC)   2    UGACACCAGGCGGA   15
```

---

6 ***yfiQ*** CP4-57 prophage; predicted protein

Energy: -13.77 p-value: 0.002

```
sRNA          21   UUACUGGGCGGC   10
                    ||||||||:|
mRNA (yfiQ)   -1   UAUGACCCGUCU   11
```

---

7 ***uhpA*** **DNA-binding response regulator in two-component regulatory system wtih UhpB**

Energy: -13.01 **p-value: 0.004**

```
sRNA          18    CUGGGCGGC     10
                     |||||||
mRNA (uhpA)  -19    UACCCGCCA    -11
```

---

8 ***yceG*** predicted aminodeoxychorismate lyase

Energy: -12.99 p-value: 0.004

```
sRNA         130    AUCGCGGGGUUAAUA    116
                     ||||||| |||||
mRNA (yceG)  -17    GAGCGCCCGAAUUAG     -3
```

---

9 ***dacD*** D-alanyl-D-alanine carboxypeptidase (penicillin-binding protein 6b)

Energy: -12.91 p-value: 0.004

```
sRNA          20    UACUGGGCGGCGGC     7
                     |||  |||||:|
mRNA (dacD)    1    UUGAAACGCCGUCU    14
```

---

10 ***csiD*** carbon starvation induced gene

Energy: -12.72 p-value: 0.005

```
sRNA          20    UAC-UGGGC-GGCGGCAG     5
                    ||| | |:| |||||||
mRNA (csiD)    4    AUGCA-CUGACCGCCGU-    20
```

---

11 ***mpaA*** murein peptide amidase A

Energy: -12.7 p-value: 0.005

```
sRNA          21    UACUGG-G--CGGCGGC     7
                    ||||||  :   |||||
mRNA (mpaA)    1    AUGACCGUAACCCGCCC    17
```

Table S2: Putative MtvR targets in the genome of *E. coli* predicted by RNAPredator

<table>
<tr><th>Energy [kJ/mol]</th><th>z-Score</th><th>Interaction</th><th>mRNA [Start]</th><th>mRNA [End]</th><th>sRNA</th><th>Gene Annotation</th></tr>
<tr><td>-14.14</td><td>-1.88</td><td>(((((((((((((.((((((((&)))))).......)))))))))))))))
mRNA: 5'- AAAUUAUGGCGCCGCC------ -3'
||||||||||||| ||
sRNA: 3'- UUUAAUAUCGCGG-GGUUAAUA -5'</td><td>-5</td><td>16</td><td>109-136</td><td>conserved protein; UPF0161 family</td></tr>
<tr><td>-13.63</td><td>-1.7</td><td>(((((((.(((((.((&)).))))).)))))))
mRNA: 5'- AGCGCCCGAAUUAGUC -3'
||||||| ||||| ||
sRNA: 3'- UCGCGGGGUUAAUAAG -5'</td><td>-16</td><td>-1</td><td>114-129</td><td><u>predicted aminodeoxychorismate lyase</u></td></tr>
<tr><td>-13.33</td><td>-1.59</td><td>(((((..(((..((((.((&))))))..)))..)))))
mRNA: 5'- GCCCCUCUUACACCUGCU -3'
||||| ||| |||| |
sRNA: 3'- CGGGGUUAAUAAGGAC-A -5'</td><td>2</td><td>20</td><td>109-126</td><td>predicted anti terminator regulatory protein</td></tr>
</table>

Table S3: Putative MtvR targets in the genome of *E. coli* predicted by sRNATarget

```
______________
    1   nfuA_b3414     predicted gluconate transport associated protein
    Probability = 1
    sRNA(sRNA1)         11 ggCGGGUCAUucuau 25
                           .|||.||||  ||
    Target(nfuA_b3414)  10 auGCCUAGUAucauu -5
______________

    2   amiA_b2435     N-acetylmuramoyl-l-alanine amidase I
    Probability = 1
    sRNA(sRNA1)         74 gACGGCCUGu 83
                           ||||||||
    Target(amiA_b2435)   1 aUGCCGGACu -9
______________

    3   gpmM_b3612     phosphoglycero mutase III, cofactor-independent
    Probability = 1
    sRNA(sRNA1)         68 cGCGGCGACg  77
                           |||.|.||
    Target(gpmM_b3612)  -1 aCGCUGUUGg -10
    Probability = 1
    sRNA(sRNA1)         71 gGCGACGGCCUg  82
                           |||||..|||
    Target(gpmM_b3612)  -1 aCGCUGUUGGAg -12
______________

    4   pmrD_b2259     polymyxin resistance protein B
    Probability = 1
    sRNA(sRNA1)         90 cGCAUCGCAa 99
                           ||||||||
    Target(pmrD_b2259)  72 uCGUAGCGUc 63
    Probability = 1
    sRNA(sRNA1)         10 cggcggGUCAUUCUAUc 26
                           |.| .|.|||||.||
    Target(pmrD_b2259)  16 acugguCGGUAAGGUAa -1
    Probability = 1
    sRNA(sRNA1)         55 uGCCGCCAGCA-cgcg 69
                           |||||||||| |||
    Target(pmrD_b2259)  79 gCGGCGGUCGUagcgu 64
______________
```

---

5 *uhpA* b3669 DNA-binding response regulator in two-component regulatory system wtih UhpB

```
Probability = 1
sRNA(sRNA1)         70 cGGCGACGGc 79
                       |||.||||
Target(uhpA_b3669)  16 cCCGUUGCCa  7
Probability = 1
sRNA(sRNA1)          3 uugacGGCGGCGG--gucau--ucuaucacccg  31
                       |.| |||..|||  .||||  |||     ||
Target(uhpA_b3669)  20 uauucCCGUUGCCacuaguaccagaaca--gga -11
```

---

6 *yfcP*_b2333 predicted fimbrial-like adhesin protein

```
Probability = 1
sRNA(sRNA1)         28 cCCGCCUGUg  37
                       ||.||.||.
Target(yfcP_b2333)  -7 aGGUGGGCAu -16
```

---

7 *fliE*_b1937 flagellar basal-body component

```
                 Probability = 1
sRNA(sRNA1)         18 cauucuaucac-ccgCCUGUGUCGCc 42
                       |..|. |||  || |||||.||||
Target(fliE_b1937)  30 uuggggaaguuagg-GGACAUAGCGa  6
```

---

8 *frsA*_b0239 hydrolase, binds to enzyme IIA(Glc)

```
Probability = 1
sRNA(sRNA1)         30 cGCCUGUGUCg 40
                       |||||||||
Target(frsA_b0239)  12 aCGGACACAGu  2
Probability = 1
sRNA(sRNA1)         102 aAGUGUUGA-----caggaa 116
                       ||.||.||     |||||
Target(frsA_b0239)   69 aUCGCAGCUccaaaguccua  50
Probability = 1
sRNA(sRNA1)          26 cACCCGCCUGu  36
                       |||||||||
Target(frsA_b0239)  -38 uUGGGCGGACu -48
Probability = 1
sRNA(sRNA1)         102 aaguguuga-----caggaauaaUUGGGGCGc 128
                       ||.||.||     |||||    ||...|||
Target(frsA_b0239)   69 aucgcagcuccaaaguccuaca-AAUUUCGCc  39
```

```
________________
    9   mntR_b0817     DNA-binding transcriptional regulator of mntH
    Probability = 1
    sRNA(sRNA1)         32 cCUGUGUCGCc 42
                           |||.|||.|
    Target(mntR_b0817)  46 uGACGCAGUGa 36
    Probability = 1
    sRNA(sRNA1)         66 cGCGCGGCGACg 77
                           |||||.||||
    Target(mntR_b0817)  15 aCGCGCUGCUGa  4
_____________
```

```
    10  relE_b1563     Qin prophage; toxin of the RelE-RelB toxin-antitoxin system
    Probability = 1
    sRNA(sRNA1)         77 gGCCUGUUUGc 87
                           |||.|.|.|
    Target(relE_b1563)  33 aCGGGCGAGCa 23
    Probability = 1
    sRNA(sRNA1)         19 auucuaucacccgccugugucgccaaaagac-GGGAAUGCcgccag 63
                                  |||.|. ||| |||||    |..|||.| ||||
    Target(relE_b1563)  42 aaggaaauca--cgggcg-agcaguuuuagguCUUUUAUG-cggua  1
_____________
```

```
    11  ymcE_b0991     cold shock gene
    Probability = 1
    sRNA(sRNA1)         85 uGCGGCGCAUc 95
                           |||||||||
    Target(ymcE_b0991)  10 uCGCCGCGUAc -1
    Probability = 1
    sRNA(sRNA1)          6 aCGGCGGCG---ggucau 20
                           |.||||||   |||||
    Target(ymcE_b0991)  12 gGUCGCCGCguaccaguc -6
_____________
```

```
    12  hybA_b2996     hydrogenase 2 4Fe-4S ferredoxin-type component
    Probability = 1
    sRNA(sRNA1)         74 gacggcCUGUUUGCGGc 90
                           |||  |||||  |||
    Target(hybA_b2996)  13 augca-GACAAGUGCCa -3
_____________
```

```
    13  apaH_b0049     diadenosine tetraphosphatase
    Probability = 1
    sRNA(sRNA1)           7 cggCGGCGGGUCa  19
                           ..|||||.|||
    Target(apaH_b0049)  -24 cuuGCCGCUCAGc -36
```

```
    Probability = 1
    sRNA(sRNA1)         33 cUGUGUCGCCAa 44
                           |.||||||||
    Target(apaH_b0049)  12 cAUACAGCGGUa  1
    Probability = 1
    sRNA(sRNA1)         36 uGUCGCCAA-aaga 48
                           |||||||| |||
    Target(apaH_b0049)  25 gCAGCGGUUauucc 12
_____________

    14  lamB_b4036     maltose outer membrane porin (maltoporin)
    Probability = 1
    sRNA(sRNA1)            7 cGGCGGCGGGuca   19
                           ||||||.||.|
    Target(lamB_b4036)  -141 aCCGCCGUCCggc -153
    Probability = 1
    sRNA(sRNA1)         15 ggUCAUUCUAUCacccgccugugucg  40
                           .||||||||||     |||| |||
    Target(lamB_b4036)   6 guAGUAAGAUAGa----ggacucagu -16
_____________

    15  srmB_b2576     ATP-dependent RNA helicase
    Probability = 1
    sRNA(sRNA1)         120 uUGGGGCGCU 129
                           |||||||||
    Target(srmB_b2576)  -14 cACCCCGCGA -23
_____________

    16  dusC_b2140     tRNA-dihydrouridine synthase C
    Probability = 1
    sRNA(sRNA1)           7 cgg-CGGCGGGUc  18
                           || |.||||.|
    Target(dusC_b2140)  -42 accaGUCGCCUAa -54
    Probability = 0.997
    sRNA(sRNA1)         22 cuAUCACCCGCc  33
                           .|||||||||
    Target(dusC_b2140)  -2 agUAGUGGGCGu -13
_____________
```

```
_________________
    17  metC_b3008     cystathionine beta-lyase, PLP-dependent
    Probability = 1
    sRNA(sRNA1)          85 ugc-GGCGCAUCg  96
                           || ||||||||
    Target(metC_b3008)  -52 gcgcCCGCGUAGg -64
    Probability = 1
    sRNA(sRNA1)         28 cccgcCUGUGUCG-----cca  43
                           ||| |..|||||     ||
    Target(metC_b3008)   8 aggc-GGUACAGCccuaagga -12
_____________

    18  lpxT_b2174     undecaprenyl pyrophosphate phosphatase
    Probability = 1
    sRNA(sRNA1)          8 gGCGGCGGGUcauucuaucac 28
                           |||||...|      ||||
    Target(lpxT_b2174)  20 aCGCCGUUUAaaaau-uagua  1
    Probability = 1
    sRNA(sRNA1)         74 gacggc-CUGUUUGCGGCg 91
                           .||..| ||.||||||||
    Target(lpxT_b2174)  32 uuguuguGAUAAACGCCGu 14
_____________

    19  hypC_b2728     protein required for maturation of hydrogenases 1 and 3
    Probability = 1
    sRNA(sRNA1)         48 aCGGGAAUGCCg 59
                           ||||||.|||
    Target(hypC_b2728)  20 gGCCCUUGCGGa  9
    Probability = 1
    sRNA(sRNA1)           5 gACGGCGGCGGGUCAuucua   24
                           |||||..|.|||||  ||
    Target(hypC_b2728)  -130 aUGCCGUUGUCCAGUu-gaa -148
_____________
```

20 *idnK*_b4268 D-gluconate kinase, thermosensitive

```
Probability = 1
sRNA(sRNA1)          87 cGGCGCAUCGCa   98
                        |||.||||.|
Target(idnK_b4268)  -93 uCCGUGUAGUGc -104
Probability = 1
sRNA(sRNA1)         24 aUCACCCGCCu 34
                       |||||||||
Target(idnK_b4268)  12 aAGUGGGCGGu  2
Probability = 0.995
sRNA(sRNA1)         66 cgCGCGGCGACg 77
                       .||||||.||
Target(idnK_b4268)  77 uuGCGCCGUUGg 66
```

---

21 *zitB*_b0752 zinc efflux system

```
Probability = 1
sRNA(sRNA1)         10 cGGCGGGUCAUUCu  23
                       |.||.|||||.|.
Target(zitB_b0752)   1 aCUGCUCAGUAGGg -13
```

---

22 *yjeS*_b4166 predicted Fe-S electron transport protein

```
Probability = 1
sRNA(sRNA1)         3 uugacggcGGCGGGUCAUu  21
                       ||||.  |.|||  |||
Target(yjeS_b4166)  7 gacugua-CUGCCUGGUAu -11
```

---

23 *ydjJ*_b1774 predicted oxidoreductase, Zn-dependent and NAD(P)-binding

```
Probability = 1
sRNA(sRNA1)         16 gucauucuaucacCCGCCUGUGUc  39
                       |||||       ||.||.||.|
Target(ydjJ_b1774)   5 aaguaaaacuua-GGUGGGCAUAa -18
```

---

24 *fliM*_b1945 flagellar motor switching and energizing component

```
Probability = 1
sRNA(sRNA1)         29 cCGCCUGUGUCGc 41
                       ||||..|||||
Target(fliM_b1945)   8 aGCGGGUACAGCa -5
```

---

25 *recE*_b1350 Rac prophage; exonuclease VIII, 5' -> 3' specific dsDNA exonuclease

```
Probability = 1
sRNA(sRNA1)         107 uUGACAGGAAuaauuggggc---gcua 130
                        |.|||||||    |||      |||
Target(recE_b1350)   28 cAUUGUCCUUcuc-accaaaacacgag   3
```

---

26 *hrpB*_b0148 predicted ATP-dependent helicase

```
Probability = 1
sRNA(sRNA1)           3 uugaCGGCGGCGGGucau 20
                        .| |.||..||||.
Target(hrpB_b0148)   26 ugccGUCGUUGCCCguu- 10
Probability = 1
sRNA(sRNA1)           4 uGACGGCGGCg 14
                        ||||||.||
Target(hrpB_b0148)   28 uCUGCCGUCGu 18

Probability = 1
sRNA(sRNA1)           6 aCGGCGGCGg 15
                        |..||.||
Target(hrpB_b0148)   13 cGUUGCUGCu  4
Probability = 1
sRNA(sRNA1)           4 ugACGGCGGC---------------gggu---cauucua  24
                        .|||.||.|               ||||   ||||||
Target(hrpB_b0148)   12 guUGCUGCUGuguuuugcgaggucucccaauuguaagaa -27
Probability = 0.999
sRNA(sRNA1)           7 cGGCGGCGGg 16
                        |.||||.|
Target(hrpB_b0148)   28 uCUGCCGUCg 19
```

---

27 *yhdH*_b3253 predicted oxidoreductase, Zn-dependent and NAD(P)-binding

```
Probability = 1
sRNA(sRNA1)         120 uUGGGGCGCuauaau 134
                         ||..|||| ||||
Target(yhdH_b3253)  -76 uACUUCGCGuuauuu -90
Probability = 0.996
sRNA(sRNA1)          19 auucuaucacc--CGCCUGUGUc 39
                        |||||       ||||||..|
Target(yhdH_b3253)   22 caagauuuucauuGCGGACGUAu -1
```

---

28 *creB*_b4398 DNA-binding response regulator in two-component regulatory system with CreC

```
Probability = 1
sRNA(sRNA1)           71 ggcg-ACGGCCUGUu   84
                         .||| |||.|||||
Target(creB_b4398)  -126 ucgcgUGCUGGACAu -140
Probability = 1
sRNA(sRNA1)         36 ugucgccaaaagacgggaaUGCCGCCA--gca  65
                        .|||||            ||||||||  ||
Target(creB_b4398)  -2 uuagcggagacaaua----ACGGCGGUgccgu -29
```

---

29 *yahD*_b0318 predicted transcriptional regulator with ankyrin domain

```
Probability = 1
sRNA(sRNA1)          9 gCGGCGGGU--------------cauuc-uaucacccg  31
                        ||||.|||              ||||| .||  |||
Target(yahD_b0318)  26 aGCCGUCCAucuaaaaaauaugaguaaguguaagggga -12
Probability = 1
sRNA(sRNA1)         22 cUAUCACCCgccugugucgccaaa 45
                        ||||||||   ||||. |||||
Target(yahD_b0318)  56 uAUAGUGGGacaacacgucgguua 33
```

---

30 ***fepB* b0592 iron-enterobactin transporter subunit**

```
Probability = 1
sRNA(sRNA1)          4 uga-cggcGGCGGGUCa 19
                        || ||| ||||.|||
Target(fepB_b0592)  19 ucucgcc-CCGCUCAGa  4
Probability = 1
sRNA(sRNA1)          46 agacgggaaugccGCCAGCACg   67
                         || |||||.   ||||||||
Target(fepB_b0592)  -98 acuccccuugu--CGGUCGUGa -117
```

---

31 *polB*_b0060 DNA polymerase II

```
Probability = 1
sRNA(sRNA1)         76 cggCCUGUUUGCGgcgca 93
                        ..||||..|||| |.|
Target(polB_b0060)  17 uuuGGACGGACGCggugc -1
Probability = 1
sRNA(sRNA1)         12 gcgggucauucuaucacccGCCUGUGUCGCc 42
                        |||||     ||  ||| |||||.|.|.|
Target(polB_b0060)  30 agcccaauucuauuuuggaCGGACGCGGUGc -1
```

```
_________________
    32  yfcS_b2336     predicted periplasmic pilus chaperone
    Probability = 1
    sRNA(sRNA1)         13 cGGGUCAUUCu---aucacc  29
                           ||.||||||.   |||||
    Target(yfcS_b2336)   1 aCCUAGUAAGgaauuagugc -19
    Probability = 1
    sRNA(sRNA1)         105 uguug--ACAGGAAUAauuggggc 126
                           |.||  ||||.||.|   ||||
    Target(yfcS_b2336)  -29 ucgacuuUGUCUUUGUc--ccccu -50
    Probability = 1
    sRNA(sRNA1)           66 cGCGCGGCGacggccuguuugc-ggcg   91
                           |||||.||   ||||   ||| |||
    Target(yfcS_b2336)  -114 cCGCGCUGCaa-cggaaauacgaccgu -139
_____________

    33  fhuE_b1102     ferric-rhodotorulic acid outer membrane transporter
    Probability = 1
    sRNA(sRNA1)         100 aAAAGUGUUgacaggaauaa-uuggg 124
                           ||||..||    |.|||   ||||
    Target(fhuE_b1102)    8 cUUUCGUAAa---cuuuagagaacca -15
_____________

    34  uhpC_b3667     membrane protein regulates uhpT expression
    Probability = 1
    sRNA(sRNA1)          91 gcaucgcaaaaaaguguuGACAGGAAu 117
                           .|| |||      |||. ||||||||
    Target(uhpC_b3667)  -41 ugu-gcgcacgg-cacguCUGUCCUUu -65
    Probability = 1
    sRNA(sRNA1)          4 ugACGGCGGCgggucauucuauca  27
                           .|||||..|  .||||.  |.|
    Target(uhpC_b3667)  12 uuUGCCGUUG--uaguaguuugga -10
    Probability = 1
    sRNA(sRNA1)         87 cGGCGCAUCG-caaaaaaguguugacaggaau 117
                           ||||||||| ||     |.|.|  |||.||.
    Target(uhpC_b3667)  36 aCCGCGUAGCcguc----cgcgaaagucuuug   9
    Probability = 1
    sRNA(sRNA1)          34 ugugucgccaaa-AGACGGGAA----------ugccgcc-agca  65
                           |.|| |||    ||||.||||          ||||.|| |||
    Target(uhpC_b3667)  -44 gcgca-cggcacgUCUGUCCUUuacacauuacacggugggucgc -86
______________
```

```
35  galF_b2042     predicted subunit with GalU
Probability = 1
sRNA(sRNA1)          15 ggucauucuaucacCCGCCUGUg  37
                        ||||||   ||| ||.|||||.
Target(galF_b2042)    6 gcaguaauaaaguaGGUGGACAu -17
```

```
36  yeaP_b1794     predicted diguanylate cyclase
Probability = 1
sRNA(sRNA1)          106 guugaca-GGAAUAAUU---gg----ggcgc 128
                         .||||| ||||.||.|   ||    ||||
Target(yeaP_b1794)     8 agacugugCCUUGUUGAgguccuaugccgcc -23
Probability = 1
sRNA(sRNA1)            8 ggCGGCGGGUc  18
                         .||||||||
Target(yeaP_b1794)   -16 auGCCGCCCAu -26
```

```
37  recQ_b3822     ATP-dependent DNA helicase
Probability = 1
sRNA(sRNA1)          79 cCUGUUUGCGgcgcau-cgca 98
                        |.|..|||| |.||| |.|
Target(recQ_b3822)   14 aGGCGGACGCgguguaagugg -7
Probability = 1
sRNA(sRNA1)          12 gcgggucauucuaucac--CCGCCUGUGUCGCc 42
                        |.|.|   ||   |||  |||||||.|.|.|
Target(recQ_b3822)   28 ggucua---aguu-gugaaGGCGGACGCGGUGu -1
```

```
38  rpiB_b4090     ribose 5-phosphate isomerase B/allose 6-phosphate isomerase
Probability = 1
sRNA(sRNA1)          16 gucauucuauca--cccgccugugu-------cgc---CAAAAGACg  50
                        |||||| |||   |||| |.|||.       |.|   |||||.||
Target(rpiB_b4090)    5 aaguaag-uagaaggggcaggcacguuuugaaguguuaGUUUUUUGu -41
```

```
39  deoC_b4381     2-deoxyribose-5-phosphate aldolase, NAD(P)-linked
Probability = 1
sRNA(sRNA1)           56 gccgcCAGCACGCg  69
                         ||| ||.|||.|
Target(deoC_b4381)   -66 aggc-GUUGUGUGa -78
Probability = 1
sRNA(sRNA1)           12 gcgggucauucua-ucacccgccug---UGUCGCCAAa  45
                         |.|.||| ||   |||    ||||   |||||||||
Target(deoC_b4381)    13 agucuagucaguaaaguaagaggaccgaACAGCGGUUa -25
```

```
_____________

    40  wzc_b2060     protein-tyrosine kinase
    Probability = 1
    sRNA(sRNA1)       15 gGUCAUUCUaucacccgc-cugug-ucg  40
                         ||||||||   ||| || |||.| ||
    Target(wzc_b2060)  6 aCAGUAAGAauauggacgagacgcaagu -22
_____________

    41  rph_b3643     defective ribonuclease PH
    Probability = 1
    sRNA(sRNA1)       71 ggcgACGGCCUGUUugcggcgcauc 95
                         || ||||||||.|   |.|||||
    Target(rph_b3643) 22 acgaUGCCGGACGAc--cugcguau -1
_____________

    42  cueR_b0487    DNA-binding transcriptional activator of copper-responsive regulon genes
    Probability = 1
    sRNA(sRNA1)        65 aCGCGCGGCGacggccuguu  84
                         |||||..||    |||||
    Target(cueR_b0487) -4 aGCGCGUUGCgga-ggacac -22
_____________


    43  yeeF_b2014    predicted amino-acid transporter
    Probability = 1
    sRNA(sRNA1)         8 ggcggcg-GGUCAUUCUa  24
                         |||.|. |||||||||
    Target(yeeF_b2014)  8 acgcuguaCCAGUAAGAg -10
_______________

    44  sdhA_b0723    succinate dehydrogenase, flavoprotein subunit
    Probability = 1
    sRNA(sRNA1)        19 auucuaucacccg-CCUGUGUCGCc  42
                         ||| ||||| |. ||..  .|.|
    Target(sdhA_b0723)  6 aaag-uagugugugGGGUGUGGUGu -18
_____________
```

45 *fliK*_b1943 flagellar hook-length control protein

```
Probability = 1
sRNA(sRNA1)         103 aguguugaca--ggaa-UAAUUGGGG-cgcuau 131
                         ||||  ||   ||   ||||  .|| |||||
Target(fliK_b1943)   42 ccacaguugcagccgccAUUAGUUCCcgcgauu  10
Probability = 1
sRNA(sRNA1)         103 aGUGUUGACa--ggaa-uaauug-gggcgcuau 131
                       ||||  ||   ||   ||||.. ||||||||
Target(fliK_b1943)   42 cCACAGUUGcagccgccauuaguucccgcgauu  10
Probability = 1
sRNA(sRNA1)         103 aguguugaca--ggaa-uaauug-GGGCGCUAu 131
                       ||||  ||   ||   ||||.. ||||||||
Target(fliK_b1943)   42 ccacaguugcagccgccauuaguuCCCGCGAUu  10
```

46 *sirA*_b3470 conserved protein required for cell growth

```
Probability = 1
sRNA(sRNA1)         47 gacgggaaugc-cgccagca-cgcgcggcgacggccuguuuGCGGCGCAucgcaaaaaaguguugacaggaauaauuggggcg 127
                       ||||   ||| |.||| || |.| |||   |||       ||.|||||  ||      |.||.||         |..||||
Target(sirA_b3470)  90 gugccaaaacgcgugguaguagug-gccaaggcc-------CGUCGCGUc-cgguucg-cgcagcucacacacc-agucccga  19
```

47 *fliO*_b1947 flagellar biosynthesis protein

```
Probability = 1
sRNA(sRNA1)         73 cgacggccuguuuGCGGCGCAU-cgca  98
                       .|||||      ||||||||| |||
Target(fliO_b1947)  -4 auugccgaguc--CGCCGCGUAagcga -28
```

48 *sgrT*_b4662 Inhibitor of glucose uptake

```
Probability = 0.999
sRNA(sRNA1)         102 aaguguugacaggaauaau---UGGGGCGCu 129
                       ||||.||| |..|        ||||||.|.
Target(sgrT_b4662)   23 aucacgacuauuuugacugcguACCCCGUGg  -8
```

49 *pcnB*_b0143 poly(A) polymerase I

```
Probability = 0.998
sRNA(sRNA1)         90 cgcaucgcaaaaaAGUGUUGA--------caggaa 116
                       .||.|       |||||..|        |||||
Target(pcnB_b0143)  -3 auguggagcca--UCACAGUUuguaguaaguccug -35
```

```
50  mscS_b2924     mechanosensitive channel
Probability = 0.995
sRNA(sRNA1)          1 -UAUUGACGGCgg-cgggucauucuauc 26
                        |||.|||..|.  |...|| |||.||
Target(mscS_b2924)  26 gAUAGCUGUUGuaaguuuag-aagguaa -1
```

```
51  mak_b0394      manno(fructo)kinase
Probability = 0.99
sRNA(sRNA1)        34 uGUGUCGCCaaaagacgggaaugccgccagcacgcgcggcgacgg-ccug  82
                       |||.|||| |||  ||   ||.||      ||||.|||  ||   |||
Target(mak_b0394)  30 cCACGGCGGauuua-gc---uauggaua---ugcgugcca-ugagaggaa -12
```

```
52  dapA_b2478     dihydrodipicolinate synthase
Probability = 0.99
sRNA(sRNA1)         67 gCGCGGCGAcgg--ccuguuugc---ggcgcauc------gcaaaaaaguguugaca-ggaauaauugg---ggcgc 128
                        ||||.|.|     ||.||  .|   ||| ||||      ||||| ||||||.     || |.  ||||   |||.
Target(dapA_b2478)  25 aGCGCUGUUaugaagggcacuuguacccg-guaggagacacguuuguucacagaguuaccaugcaaaccauaccgua -51
```

Table S4: regions with homology to within the genome of *E. coli*

```
1- (2662273..2662285) non-coding region between suhB and yfhR

 Score = 26.3 bits (13), Expect = 7.0
 Identities = 13/13 (100%)
 Strand = Plus / Minus

Query: 23      tatcacccgcctg 35
               |||||||||||||
Sbjct: 2662285 tatcacccgcctg 2662273
_____________

2- (2519454..2519466) non-coding region between lysV and REPv173

 Score = 26.3 bits (13), Expect = 7.0
 Identities = 13/13 (100%)
 Strand = Plus / Plus

Query: 68      cgcggcgacggcc 80
               |||||||||||||
Sbjct: 2519454 cgcggcgacggcc 2519466
_____________

3- (780979..780991) non-coding region between lysQ and nadA

 Score = 26.3 bits (13), Expect = 7.0
 Identities = 13/13 (100%)
 Strand = Plus / Plus

Query: 68     cgcggcgacggcc 80
              |||||||||||||
Sbjct: 780979 cgcggcgacggcc 780991

_____________

3- (3325770..3325781) non-coding region between rlmE and yhbY

 Score = 24.3 bits (12), Expect =    28
 Identities = 12/12 (100%)
 Strand = Plus / Minus

Query: 13      cgggtcattcta 24
               ||||||||||||
Sbjct: 3325781 cgggtcattcta 3325770

_____________

5- (1877381..1877392) non-coding region between yeaQ and yoaG

 Score = 24.3 bits (12), Expect =    28
 Identities = 12/12 (100%)
 Strand = Plus / Minus

Query: 92      catcgcaaaaaa 103
               ||||||||||||
Sbjct: 1877392 catcgcaaaaaa 1877381
_____________
```

6- (1500400..1500411) non-coding region between *tehB* and *ydcL*

```
 Score = 24.3 bits (12), Expect =    28
 Identities = 12/12 (100%)
 Strand = Plus / Minus

Query: 98      aaaaaagtgttg 109
               ||||||||||||
Sbjct: 1500411 aaaaaagtgttg 1500400
```

___

7- (1405859..1405870) non-coding region between *ydaM* and *ydaN*

```
 Score = 24.3 bits (12), Expect =    28
 Identities = 12/12 (100%)
 Strand = Plus / Plus

Query: 93      atcgcaaaaaag 104
               ||||||||||||
Sbjct: 1405859 atcgcaaaaaag 1405870
```

___

8- (1314409..1314420) non-coding region between *yciG* and *trpA*

```
 Score = 24.3 bits (12), Expect =    28
 Identities = 12/12 (100%)
 Strand = Plus / Plus

Query: 53      aatgccgccagc 64
               ||||||||||||
Sbjct: 1314409 aatgccgccagc 1314420
```

___

9- (741938..741949) non-coding region between *dtpD* and *ybgI*

```
 Score = 24.3 bits (12), Expect =    28
 Identities = 12/12 (100%)
 Strand = Plus / Minus

Query: 39     cgccaaaagacg 50
              ||||||||||||
Sbjct: 741949 cgccaaaagacg 741938
```

Table S5: Putative MtvR targets in the genome of *P. aeruginosa* UCBPP-PA14 predicted by TargetRNA

---

1 <u>PA14_15920</u> major facilitator transporter

Energy: -21.91 p-value: 0.000

```
sRNA                 21   UUACUGGGCGGCGGC   7
                           |||| ||||||||
mRNA (PA14_15920)    -1   CAUGA-CCGCCGCCA   13
```

---

2 <u>PA14_39850</u> MFS transporter

Energy: -21.71 p-value: 0.000

```
sRNA                 21   UAC---U-GGGCGGCGGCAGU   4
                          |||   | ||||||||| ||
mRNA (PA14_39850)    1    AUGACCACCCCGCCGCCCUC-   20
```

---

3 <u>katA</u> catalase

Energy: -21.03 p-value: 0.000

```
sRNA                 24   AUCUUACUGGGCGGC   10
                           |||| ||||||||
mRNA (katA)          7    GAGAA-GACCCGCCU   20
```

---

4 <u>fepB</u> iron-enterobactin transporter periplasmic binding protein

Energy: -20.33 p-value: 0.000

```
sRNA                 19   ACUGGGCGGCGGCA   6
                           ||||||||||||
mRNA (fepB)          5    CGACCCGCCGCCGC   18
```

---

5 <u>PA14_69340</u> ABC transporter ATP-binding protein

Energy: -20.29 p-value: 0.000

```
sRNA                 17   UGGGCGGCGGC   7
                           :||||||||
mRNA (PA14_69340)    -10  CUCCGCCGCCA   1
```

---

6 <u>PA14_18050</u> hypothetical protein

Energy: -20.18 p-value: 0.000

```
sRNA                 17   UGGGCGGCGGCA   6
                           ||||||||||
mRNA                 -20  CCCCGCCGCCGA   -9
```

(PA14_18050)

---

7 **PA14_69880** LysR family transcriptional regulator

Energy: -19.74 p-value: 0.000

sRNA	22	`CUUACUGGGCGGCGGC`	7
		` |||||||||:||||`	
mRNA (PA14_69880)	-2	`CAAUGACCCGUCGCCU`	14

---

8 **modA** molybdate-binding periplasmic protein precursor modA

Energy: -18.98 p-value: 0.000

sRNA	21	`UUACUGG-GCGGCGGC`	7
		` |||||| | |||||`	
mRNA (*modA*)	-1	`CAUGACCAC-CCGCCU`	14

---

9 **ureD** urease accessory protein

Energy: -18.81 p-value: 0.000

sRNA	21	`UUACUGGGCGGCGGCAGU`	4
		`:|||| |||||||| ||`	
mRNA (*ureD*)	-2	`GAUGA-CCGCCGCCCUCC`	16

---

10 **PA14_56960** hypothetical protein

Energy: -18.31 p-value: 0.000

sRNA	20	`UACUGGG--CGGCGGCAGU`	4
		`|||||||  |||||||||`	
mRNA (PA14_56960)	1	`AUGACCCAGGCCGCCGUCC`	19

---

11 **gacA** **response regulator GacA**

Energy: -17.85 p-value: 0.000

sRNA	71	`GC--G--CGCACGACCGCCGU`	55
		`:|  :  | |||||||:||:`	
mRNA (***gacA***)	1	`UGAUUAAG-GUGCUGGUGGU-`	20

---

12 **PA14_53090** transcriptional regulator

Energy: -17.85 p-value: 0.000

sRNA	19	`ACUGGGCGGCGGC`	7
		` |||||||| ||`	
mRNA (PA14_53090)	7	`CGACCCGCC-CCA`	18

---

13 PA14_12610 transporter

Energy: -17.78 p-value: 0.000

```
sRNA                 130   AUCGCGGGGUU   120
                            |||||||||
mRNA (PA14_12610)     -9   AAGCGCCCCAU     2
```

14 PA14_28980 Fe^{2+}-dicitrate sensor

Energy: -17.7 p-value: 0.000

```
sRNA                  21   UACUGGGCGGCGGC   7
                           |||||||||:|||
mRNA (PA14_28980)    -20   AUGACCCGCUGCCU  -7
```

15 fadH1 2,4-dienoyl-CoA reductase

Energy: -17.64 p-value: 0.000

```
sRNA                  20   UACUGGGCGGCGGCAGU   4
                           |||| ||||||||||:
mRNA (fadH1)          -1   AUGA-CCGCCGCCGUUC  16
```

16 PA14_53920 transcriptional regulator

Energy: -17.63 p-value: 0.000

```
sRNA                  21   UACUGGG-C---GGCGGC    7
                           ||||||| |   |||||
mRNA (PA14_53920)      1   AUGACCCAGAACCCGCCC   18
```

17 PA14_18060 hypothetical protein

Energy: -17.58 p-value: 0.000

```
sRNA                  18   CUGGGCGGCGGC    7
                            |:|||||||:
mRNA (PA14_18060)      6   CAUCCGCCGCUA   17
```

18 aceK bifunctional isocitrate dehydrogenase kinase/phosphatase

Energy: -17.53 p-value: 0.000

```
sRNA                 130   AUCGCGGGGUUA   119
                            |||||||||:
mRNA (aceK)            9   GAGCGCCCCAGC    20
```

19 **PA14_15280** hypothetical protein

Energy: -16.95 p-value: 0.000

```
sRNA              21   UUACUGGGCGGCGG   8
                        ||||||| ||||
mRNA
(PA14_15280)      -1   CAUGACCCACCGCA   13
```

---

20 **PA14_20491** acetyltransferase

Energy: -16.87 p-value: 0.000

```
sRNA              83   UGUCCGGCAGCGGC   70
                        ||| ||||||||
mRNA
(PA14_20491)      6    CCAGACCGUCGCCA   19
```

---

21 **PA14_50850** hypothetical protein

Energy: -16.5 p-value: 0.000

```
sRNA              23   UCUUACUGGGCGGC   10
                        ||||||||| ||
mRNA
(PA14_50850)      -3   CGAAUGACCC-CCU   10
```

---

22 **PA14_22280** pirin-related protein

Energy: -16.39 p-value: 0.000

```
sRNA              21   UUACUGGGCGGCGGCA   6
                        |||||||||: ||||
mRNA
(PA14_22280)      -1   CAUGACCCGCUACCGU   15
```

---

23 **PA14_44770** guanine deaminase

Energy: -16.34 p-value: 0.000

```
sRNA              21   UUACUGGGCG--GCGGC   7
                        |||||||||  ||||
mRNA
(PA14_44770)      -1   CAUGACCCGCAACGCC-   15
```

---

24 **PA14_29090** periplasmic spermidine/putrescine-binding protein

Energy: -15.88 p-value: 0.000

```
sRNA              23   UCUUACUGGGCG--GCGGC   7
                        ||| |||||||  |||:
mRNA
(PA14_29090)      2    UGAA-GACCCGCAUCGCUU   19
```

25 **PA14_40280** hypothetical protein

Energy: -15.57 p-value: 0.000

```
sRNA              23   UCUU--ACUGGGCGGCGGCA   6
                        ||:  |  ||| ||||||
mRNA (PA14_40280)  2   UGAGCCUUUCCC-CCGCCGA   20
```

26 **PA14_31070** hypothetical protein

Energy: -15.57 p-value: 0.000

```
sRNA              77   GCAGCGGCGCGCACGACCGCC   57
                       :||||:|||||||||  |: |
mRNA (PA14_31070)  2   UGUCGUCGCGCGUGC-AGU-G   20
```

27 **fepC** **ferric enterobactin transport protein FepC**

Energy: -15.5 p-value: 0.000

```
sRNA         21   UUACUGGGCGGCGGC   7
                   ||||||| :||||
mRNA (fepC)  -1   CAUGACCCAUCGCCU   14
```

28 **PA14_38950** hypothetical protein

Energy: -15.49 p-value: 0.000

```
sRNA              18   CUGGGC-GGCGGCAG   5
                        :|||| |||||||
mRNA (PA14_38950)  2   UGCCCGACCGCCGUA   16
```

29 **pykA** pyruvate kinase

Energy: -15.45 p-value: 0.000

```
sRNA         81   UCCGGC-AGCGGCGC   68
                   | ||| |||||||
mRNA (pykA)   2   UGUCCGUUCGCCGCA   16
```

30 **phaD** poly(3-hydroxyalkanoic acid) depolymerase

Energy: -15.26 p-value: 0.001

```
sRNA          15   GCGGCGGCA   6
                   ||||||||
mRNA (phaD)  -20   CGCCGCCGA   -12
```

31 **PA14_29760** chemotaxis transducer

Energy: -15.25 p-value: 0.001

```
sRNA               17   UGGGCGGCGG-CAGU   4
                         ::||||||| |||
mRNA (PA14_29760)   7   CUUCGCCGCCUGUC-   20
```

32 **PA14_26350** hypothetical protein

Energy: -15.08 p-value: 0.001

```
sRNA               75   AGCGGCGCGC   66
                         ||||||||
mRNA (PA14_26350)  -20  ACGCCGCGCA   -11
```

33 **algF** alginate o-acetyltransferase AlgF

Energy: -15.01 p-value: 0.001

```
sRNA               22   CUUACUGGGCGGCG    9
                         :|||||||||||
mRNA (algF)         8   CGAUGACCCGCCG-    20
```

34 **PA14_15190** hypothetical protein

Energy: -15 p-value: 0.001

```
sRNA               17   UGGGCGGCGGCAGUU   3
                         |||  :|||||||
mRNA (PA14_15190)  -4   UCCC-AUGCCGUCAU   10
```

35 **PA14_43650** hypothetical protein

Energy: -14.84 p-value: 0.001

```
sRNA               76   CAGCGGCGCGCACGA   62
                         ||||: |||||||
mRNA (PA14_43650)   7   AUCGCUUCGCGUGC-   20
```

36 **PA14_17990** hypothetical protein

Energy: -14.83 p-value: 0.001

```
sRNA           21   UUACUGGGCGGCGGC    7
                     ||||||| |:|||
mRNA
(PA14_17990)   -1   CAUGACCC-CUGCCA   13
```

---

37 **PA14_53210** hypothetical protein

Energy: -14.56 p-value: 0.001

```
sRNA           19   ACUGGGCGGCGGCA    6
                     || |||||||||
mRNA
(PA14_53210)    7   GGAACCGCCGCCGA   20
```

---

38 **PA14_03490** hypothetical protein

Energy: -14.47 p-value: 0.001

```
sRNA           21   UUACUGG-GCGGCGGCAG-U    4
                     |||||| | ||||| || |
mRNA
(PA14_03490)   -1   CAUGACCAC-CCGCC-UCGA   17
```

---

39 **PA14_46360** two-component response regulator

Energy: -14.41 p-value: 0.001

```
sRNA           24   AUCUUACUGGGCGGCG    9
                     ||||||||: ||||
mRNA
(PA14_46360)   -4   AAGAAUGACUGGCCG-   11
```

---

40 **PA14_15720** transglycosylase

Energy: -14.25 p-value: 0.001

```
sRNA           71   GCGCGCACGACCGCCG   56
                     |||||||| |  ||
mRNA
(PA14_15720)   -5   AGCGCGUGCAGAAGG-   10
```

---

41 **pilF** type 4 fimbrial biogenesis protein PilF

Energy: -14.24 p-value: 0.001

```
sRNA           78   GGCAGCGGCGCGC   66
                     || ||||||||
mRNA (pilF)     9   ACG-CGCCGCGCU   20
```

---

42 **recO** DNA repair protein RecO

Energy: -14.18 p-value: 0.001

```
sRNA           19    ACUGGGCGGCGGCAGU     4
                      ||||||||  :|||
mRNA (recO)    -9    CGACCCGCC-AUGUCC     6
```

---

43 **PA14_20880** hypothetical protein

Energy: -14.12 p-value: 0.002

```
sRNA                17    UGGG---CGGCGGCAGUU     3
                           |||   ||||||| ||
mRNA (PA14_20880)   -16   CCCCUGAGCCGCCGACAU     2
```

---

44 **PA14_26260** hydrolase

Energy: -14.03 p-value: 0.002

```
sRNA                27    ACUAUCUUACUGG-GCGGC    10
                           || |||||||||  |||
mRNA (PA14_26260)   -6    AGA-AGAAUGACCGAGCCU    12
```

---

45 **nosF** NosF protein

Energy: -13.92 p-value: 0.002

```
sRNA           17    GGGCGGCGGCA     6
                     |||||||| |
mRNA (nosF)    -20   CCCGCCGCGGA     -10
```

---

46 **PA14_11650** hypothetical protein

Energy: -13.81 p-value: 0.002

```
sRNA                17    GGGCGGCGGCAGUUA-     2
                          |||||||||  ||:|
mRNA (PA14_11650)   -20   CCCGCCGCC--CAGUG     -7
```

---

47 **PA14_12670** hypothetical protein

Energy: -13.74 p-value: 0.002

```
sRNA                66    CACG-ACCGCCGUAAGGGC    49
                           |||  |||||||  |:|
mRNA (PA14_12670)   1     AUGCGCGGCGGCAAGCUCU    19
```

---

48 **PA14_49720** hypothetical protein

Energy: -13.64 p-value: 0.002

```
sRNA               20     UACUG-GGCGGCGGCAG    5
                          ||| | |:||||||||
mRNA (PA14_49720)  1      AUGUCACUGCCGCCGUA    17
```

49 **PA14_68920** LysR family transcriptional regulator

Energy: -13.57 p-value: 0.002

```
sRNA               19     ACUGG--GCGGCGGC    7
                           ||||  |||||||
mRNA (PA14_68920)  6      CGACCUACGCCGCCU    20
```

50 **PA14_40650** hypothetical protein

Energy: -13.31 p-value: 0.003

```
sRNA               23     UCUUAC-UGGGCGGCGGC    7
                           |:| | ||||||| ||
mRNA (PA14_40650)  -15    CGGA-GAACCCGCC-CCA    1
```

51 **PA14_46310** hypothetical protein

Energy: -13.25 p-value: 0.003

```
sRNA               23     UCUUACUGGGCGGC-GGCA    6
                           | |||||||| :| |||
mRNA (PA14_46310)  -3     CGCAUGACCCG-UGACCG-    14
```

52 **PA14_42920** hypothetical protein

Energy: -13.18 p-value: 0.003

```
sRNA               39     CUGUGUCCGCCCACUA    24
                          |:|| ||||||||||
mRNA (PA14_42920)  -20    GGCA-AGGCGGGUGAC    -6
```

53 **PA14_29210 MFS transporter**

Energy: -13.17 p-value: 0.003

```
sRNA               19     AC-UGGGCGGCGGC    7
                           | ||||||||||
mRNA (PA14_29210)  6      CGCACCCGCCGCCU    19
```

54 **PA14_37910** LysR family transcriptional regulator

Energy: -13.14 p-value: 0.003

```
sRNA           19   ACUGGGC-GGCGGCAGUUA    2
                     ||||:|  :||||||||
mRNA
(PA14_37910)    3   GGACCUGAAUGCCGUCAA-   20
```

55 **PA14_58440 ABC transporter permease**

Energy: -13.12 p-value: 0.003

```
sRNA           24   AUCUUACUGGGCGGC   10
                     :||:| |||||||
mRNA
(PA14_58440)  -17   AGGAGUUACCCGCCA   -3
```

56 **PA14_16740** hypothetical protein

Energy: -13.11 p-value: 0.003

```
sRNA           19   ACU-GGGCGGCGGC   7
                     || |||||||||
mRNA
(PA14_16740)  -13   GGAGCCCGCCGCCA   1
```

57 **PA14_05040** coproporphyrinogen III oxidase

Energy: -13.1 p-value: 0.003

```
sRNA           17   UGGGC--GGCGGCAGUU   3
                     ||||  ||||||| |
mRNA
(PA14_05040)  -12   CCCCGCCCCGCCGUGAC   5
```

58 **PA14_71100** hypothetical protein

Energy: -13.09 p-value: 0.003

```
sRNA           18   CUGGGCGGCGGCAGUUA    2
                     |||| || |||||||
mRNA
(PA14_71100)    6   CACCCUCC-CCGUCAA-   20
```

59 **napF** ferredoxin protein NapF

Energy: -13.02 p-value: 0.004

```
sRNA           80   CCGGCAGCGG--CGCGC   66
                     || ||||||  | ||
mRNA (napF)     4   AGCAGUCGCCGAGAGCU   20
```

60 **PA14_06460** hypothetical protein

Energy: -12.99 p-value: 0.004

```
sRNA               75     GCGGCGCGC           66
                          ||||||||
mRNA (PA14_06460)  -20    CGCCGCGCA           -12
```

61 **adhA** alcohol dehydrogenase

Energy: -12.97 p-value: 0.004

```
sRNA               27     CUAUCU--UACUGGG-CGGCGG    8
                          ||| ||  ||||||| ||| |
mRNA (adhA)        -7     GAU-GACCAUGACCCUGCCACA    14
```

62 **PA14_61990** hypothetical protein

Energy: -12.84 p-value: 0.004

```
sRNA               19     ACUGGGCGGCGGCA      6
                           |||||||:  ||
mRNA (PA14_61990)  8      AGACCCGCU-ACGG      20
```

63 **PA14_60550** ATP-dependent protease

Energy: -12.77 p-value: 0.004

```
sRNA               79     CGGCAGCGGCGC        68
                           |||||||:||
mRNA (PA14_60550)  10     UCCGUCGCUGC-        20
```

64 **PA14_58110** Maf-like protein

Energy: -12.65 p-value: 0.005

```
sRNA               83     UGUCCGGCAGCGGCGC    68
                           || ||||||||:|:
mRNA (PA14_58110)  -2     CCAUGCCGUCGCUGUA    14
```

Table S6: Putative MtvR targets in the genome of *P. aeruginosa* UCBPP-PA14 predicted by RNAPredator

<table>
<tr><th>Energy [kJ/mol]</th><th>z-Score</th><th>Interaction</th><th>mRNA [Start]</th><th>mRNA [End]</th><th>sRNA</th><th>Gene Annotation</th></tr>
<tr><td>-16.06</td><td>-2.49</td><td>(((.(.(((((((..(((((((((&))))))))))))))))).).)))
mRNA: 5'- GAUCGCAUGACCCAGGCCGCCG -3'
||| | ||||||| |||||||
sRNA: 3'- CUAUCUUACUGGG--CGGCGGC -5'</td><td>-6</td><td>17</td><td>5-26</td><td>hypothetical protein 56960</td></tr>
<tr><td>-16.04</td><td>-2.48</td><td>((((((...(((((((((((&)))))))))))..))))))
mRNA: 5'- GAUGGACUGGACCCGCCGC -3'
|||||| ||||||||||
sRNA: 3'- CUAUCUUA-CUGGGCGGCG -5'</td><td>-1</td><td>18</td><td>8-26</td><td>transcriptional regulator</td></tr>
<tr><td>-15.91</td><td>-2.43</td><td>(((((((..((.(((((((.((((((&))))))))))))).))..)))))))
mRNA: 5'- GCGGGUGCCGGCAUGACCCAGCCGC -3'
||||||| || ||||||| |||||
sRNA: 3'- CGCCCACUAUCUUACUGGG-CGGCG -5'</td><td>-12</td><td>13</td><td>8-32</td><td>hypothetical protein 34940</td></tr>
<tr><td>-15.49</td><td>-2.28</td><td>(((((....(((((((((..(((((((&)))))))..))).....)))))))))))
mRNA:5'- GUAGCCCAUGC-----CCCA-UCCCAU -3'
||||| || || ||||||
sRNA:3'- UAUCG----CGGGGUUAA-UAAGGACA -5'</td><td>-6</td><td>20</td><td>104-131</td><td>putative hydrolase</td></tr>
<tr><td>-15.39</td><td>-2.24</td><td>((((((.((.((((((((((((&)))))))))))))))).))))
mRNA: 5'- CGUG-GCAUGAGCGGCAUUCC -3'
|||| | || |||||||||||
sRNA: 3'- GCGCAC-GA-CCGCCGUAAGG -5'</td><td>-5</td><td>16</td><td>49-69</td><td>phenazine biosynthesis protein **PhzD**</td></tr>
</table>

<table>
<tr><th>Energy [kJ/mol]</th><th>z-Score</th><th>Interaction</th><th>mRNA [Start]</th><th>mRNA [End]</th><th>sRNA</th><th>Gene Annotation</th></tr>
<tr><td>-14.54</td><td>-1.94</td><td>(((((((((....(((&))).....)))))))))
mRNA: 5'- AGCGCCCAUGAG-UC -3'
          ||||||||| ||
sRNA: 3'- UCGCGGGGUUAAUAAG -5'</td><td>-7</td><td>8</td><td>113-129</td><td>putative transporter</td></tr>
<tr><td>-14.39</td><td>-1.88</td><td>((((((((((((&))))))).....)))))
mRNA: 5'- CCCCA-----UC -3'
          ||||| ||
sRNA: 3'- GGGGUUAAUAAG -5'</td><td>-13</td><td>-2</td><td>109-125</td><td>hypothetical protein 70860</td></tr>
<tr><td>-14.27</td><td>-1.84</td><td>((.(((((.((((((.(((((((&)))))))..)))))).)))))))
mRNA: 5'- GGAGUGA-UUGAAU-GAAUGCCG -3'
          || |||| ||||| |||||||
sRNA: 3'- CC-CACUA-UCUUACUGGGCGGC -5'</td><td>-3</td><td>19</td><td>8-30</td><td>endonuclease III</td></tr>
<tr><td>-14.22</td><td>-1.82</td><td>((((((((((((.((&))))))))))))))
mRNA: 5'- GACCCGCCGCCGCU -3'
          |||||||||||| |
sRNA: 3'- CUGGGCGGCGGC-A -5'</td><td>6</td><td>20</td><td>5-18</td><td>iron enterobactin transporter periplasmic binding protein</td></tr>
</table>

<table>
<tr><th>Energy [kJ/mol]</th><th>z-Score</th><th>Interaction</th><th>mRNA [Start]</th><th>mRNA [End]</th><th>sRNA</th><th>Gene Annotation</th></tr>
<tr><td>-13.54</td><td>-1.57</td><td>(((((((((((((&)))))))).....)))))<pre>
mRNA: 5'- GCCCU-----UUC -3'
          ||||| |||
sRNA: 3'- CGGGGUUAAUAAG -5'
</pre></td><td>-11</td><td>1</td><td>109-126</td><td>3 dehydroquinate synthase</td></tr>
<tr><td>-13.29</td><td>-1.48</td><td>(((((((((((&)))))))))))<pre>
mRNA: 5'- CGAUGCGCCGC -3'
          |||||||||||
sRNA: 3'- GCUACGCGGCG -5'
</pre></td><td>-2</td><td>8</td><td>86-96</td><td>hypothetical protein</td></tr>
<tr><td>-13.11</td><td>-1.42</td><td>(((((..((.((.(((((((((((((((((&))).)))))))))))))).)).)).)))))<pre>
mRNA: 5'- GCGGGAGG-AA-GGCAUGAUCCGCUG-UCG -3'
          ||||| | | ||||||||||||| |||
sRNA: 3'- CGCCCA-CU-AU-CUUACUGGGCGGCGGCA -5'
</pre></td><td>-13</td><td>16</td><td>3-32</td><td>hypothetical protein 38270</td></tr>
<tr><td>-13.05</td><td>-1.40</td><td>((.(((((((((((.(((..(((((.((&)).)))))))).))))))).))))))<pre>
mRNA: 5'- GGAGUG-AAGAAUGAGCCGUACC-GC -3'
           || ||| ||||||| ||| ||| |
sRNA: 3'- CC-CACUAUCUUACUGGG--CGGCG- -5'
</pre></td><td>-10</td><td>17</td><td>5-30</td><td>putative secreted lipoprotein</td></tr>
</table>

Supplementary References

1. Markowitz VM, Chen IMA, Palaniappan K, Chu K, Szeto E, et al. (2010) The integrated microbial genomes system: an expanding comparative analysis resource. Nucleic Acids Res 30: D382-D390.
2. Zhao Y, Li H, Hou Y, Cha L, Cao Y, et al. (2008) Construction of two mathematical models for prediction of bacterial sRNA targets. Biochem Biophys Res Commun 372: 346-350.
3. Tjaden B, Goodwin SS, Opdyke JA, Guillier M, Fu DX, et al. (2006) Target prediction for small, noncoding RNAs in bacteria. Nucleic Acids Res 34: 2791-2802.
4. Eggenhofer F, Tafer H, Stadler PF, Hofacker IL (2011) RNApredator: fast accessibility-based prediction of sRNA targets. Nucleic Acids Res 39: W149-W154.
